# The compound LY295427 antagonizes 25-hydroxycholesterol through binding to INSIG

**DOI:** 10.1016/j.jlr.2026.101015

**Published:** 2026-03-06

**Authors:** Xing-Yan Wen, De-Jie Zhang, Li-Ming He, Ming Sun, Qi Jiang, Ying Zhu, Bao-Liang Song, Wen-Wei Qiu, Xiao-Yi Lu

**Affiliations:** 1 State Key Laboratory of Metabolism and Regulation in Complex Organisms, College of Life Sciences, Taikang Center for Life and Medical Sciences, Wuhan University, Wuhan, China; 2 Shanghai Engineering Research Center of Molecular Therapeutics and New Drug Development, School of Chemistry and Molecular Engineering, East China Normal University, Shanghai, China; 3 Department of Epidemiology and Biostatistics, School of Public Health, Wuhan University, Wuhan, China

**Keywords:** cholesterol, 25-HC, HMGCR, INSIG, LY295427

## Abstract

25-Hydroxycholesterol (25-HC) regulates cholesterol metabolism by inhibiting the maturation of SREBP and promoting the degradation of 3-hydroxy-3-methylglutaryl-CoA reductase (HMGCR). The compound LY295427 can reverse 25-HC-mediated suppression of SREBP processing, but the mechanism is unclear. In addition, it is unknown whether LY295427 is able to antagonize the sterol-regulated degradation of HMGCR. In this study, we found that LY295427 prevented the 25-HC-induced interaction between SREBP cleavage-activating protein and INSIG-1 and caused the translocation of SREBP cleavage-activating protein to the Golgi even in the presence of 25-HC. Using a photoreactive LY295427 probe, we demonstrated that it directly bound to INSIG-1, which could be competed off by 25-HC. In addition, LY295427 blocked 25-HC-induced ubiquitination and degradation of HMGCR. Together, this study suggests that LY295427 competes with 25-HC to bind INSIG and therefore blunts 25-HC-induced inhibition of SREBP processing and degradation of HMGCR.

Cholesterol is a crucial lipid molecule in mammalian cells, regulating the rigidity, fluidity, and permeability of the cell membrane. Additionally, it serves as a precursor for bile acids and steroids. Abnormal metabolism of cholesterol has been associated with cardiovascular disease and Niemann-Pick type C disease (1). Mammalian cells obtain cholesterol through two primary mechanisms: endocytosis of lipoproteins and de novo synthesis in the endoplasmic reticulum (ER). The sterol-regulated degradation of 3-hydroxy-3-methylglutaryl-CoA reductase (HMGCR) and the proteolytic cleavage of SREBP are two major negative feedback mechanisms that control intracellular cholesterol level (2, 3, 4, 5). When sterol levels in the ER are low, SREBP cleavage-activating protein (SCAP) transfers SREBP from the ER to the Golgi apparatus. There, SREBP is cleaved by proteases, liberating its N-terminal domain that is then transported into the nucleus to upregulate cholesterol synthesis genes (6). Conversely, when sterol levels in the ER are high, SCAP undergoes a conformational change and binds to the ER-resident protein INSIG, forming the INSIG-SCAP-SREBP complex. This complex stays in the ER, inhibiting the cleavage of SREBP and subsequently downregulating the expression of cholesterol synthesis genes and LDL receptor (LDLR) (7). High levels of sterols in the ER can also accelerate the ubiquitination and degradation of HMGCR, the rate-limiting enzyme in the cholesterol biosynthesis pathway. Sterol promotes the interaction between HMGCR and INSIG, which further recruits the E3 ubiquitin ligases, including gp78, TRC8, or RNF145. Then HMGCR is ubiquitinated and degraded. This process ultimately decreases the rate of cholesterol synthesis (8, 9, 10, 11, 12, 13, 14).

25-Hydroxycholesterol (25-HC) can potently inhibit SREBP processing and accelerate HMGCR degradation. By binding directly to the INSIG protein or INSIG-SCAP complex (4, 15), 25-HC enhances the INSIG-SCAP interaction and suppresses SREBP processing. Although direct structural evidence is lacking, it is speculated that 25-HC binds INSIG and then promotes HMGCR degradation (16).

The sterol LY295427 is identified through a screen for compounds that reverse 25-HC-mediated suppression of the LDLR promoter reporter gene (17). LY295427 shows cholesterol-lowering activity that upregulates the expression of the LDLR gene in hypercholesterolemic hamsters and rabbits (18). LY295427 lowers cholesterol by more than 70% in hamsters fed coconut oil, but it has no effect in normal chow-fed hamsters. As many lipogenic genes are upregulated besides LDLR, the clinical application of LY295427 is unclear. Previous studies have demonstrated that LY295427 can antagonize the ability of 25-HC to inhibit SREBP processing (19). The molecular mechanism of LY295427 antagonizing 25-HC has long remained unexplained.

In this study, we demonstrate that LY295427 can reverse the inhibition of SREBP2 by 25-HC through binding to INSIG. We further find that LY295427 can antagonize 25-HC-induced degradation of HMGCR.

## Materials and Methods

### Reagents

Mevalonate (#4667), 25-HC (#700019P), anti-c-Myc agarose beads (#E6654), anti-FLAG M2 agarose beads (#A2220), *N*-ethylmaleimide (#E3876), sodium L-ascorbate (#A7631), biotin-N3 (#762024), CuSO_4_ (#C8027), PMSF (#P7626), and cocktail (#P8340) were obtained from Sigma-Aldrich. Lovastatin (purity >98.5%, as determined by HPLC) was acquired from Shanghai Pharm Valley, and TBTA (#T2993) was obtained from Tokyo Chemical Industry. MG132 (#I-130), ubiquitin-activating enzyme UBE1 (E1), and FLAG-ubiquitin (#U-120-01M) were sourced from Boston Biochem. Puromycin (BS111) was obtained from Biosharp. G418 (#345810), pepstatin A (#516481), and *N*-acetyl-leucyl-leucyl-norleucinal (#208719) were obtained from Calbiochem. Leupeptin (#11034626001) was obtained from Roche. DL-DTT (#A100281), ATP (#A600311), and NP-40 (#A100109) were obtained from Sangon Biotech. Cycloheximide (CHX, #HY-12320) was obtained from MCE. LY295427 and LY295427 probe (purity >98%, determined by HPLC) were generously provided by Professor Wen-Wei Qiu from East China Normal University, Shanghai. Lipoprotein-deficient serum (LPDS, density >1.215 g/ml) was prepared from newborn calf serum by ultracentrifugation in our laboratory. Ubiquitin-conjugating enzyme UBE2G2 (E2, Ubc7) was homemade by *Escherichia coli* expression.

### Antibodies

The primary antibodies used were as follows: mouse monoclonal antibody against β-actin (#A5441; Sigma), mouse monoclonal antibody against FLAG-tag (#F3165; Sigma), mouse monoclonal antibody against T7-tag (#69522; Merck), mouse monoclonal antibody against HA-tag (#H3663; Sigma), mouse monoclonal antibody P4D1 against ubiquitin (#SC-8017; Santa Cruz Biotechnology), mouse monoclonal antibody 9D5 against SCAP (#CRL-2347; ATCC), mouse monoclonal antibody 7D4 against SREBP2 (#CRL-2198; ATCC), anti-farnesyl-diphosphate farnesyltransferase 1 (FDFT1) antibody (#13128; Proteintech), anti-lanosterol synthase (LSS) antibody (#18693; Proteintech), mouse monoclonal antibody 9E10 against MYC-tag, mouse monoclonal antibody A9 against HMGCR, mouse monoclonal antibody 1D2 against SREBP2, rabbit polyclonal antibody against enhanced GFP (EGFP)-tag, rabbit polyclonal antibody against LDLR, and rabbit polyclonal antibody against HMGCR, which were prepared in our laboratory. The secondary antibodies used were as follows: peroxidase AffiniPure goat anti-mouse IgG (H + L) secondary antibody (#115-035-003; Jackson ImmunoResearch), peroxidase AffiniPure goat anti-rabbit IgG (H + L) secondary antibody (#111-035-144; Jackson ImmunoResearch), Alexa Fluor 555 donkey anti-mouse IgG (#A31570; Invitrogen), and mouse monoclonal antibody against GM130 (#610822; BD Biosciences).

### Plasmids

pLVX-HMGCR (TM1-8)-EGFP was constructed by inserting the nucleotide fragment corresponding to amino acids 1 to 346 of hamster HMGCR into pLVX–IRES–Neo (Clontech), and this was confirmed by sequencing. The constructs pCMV-HMGCR-T7, pCMV-HMGCR-FLAG, pCMV-INSIG-1-Myc, pCMV-INSIG-2-Myc, pEF-HA-Ubiquitin, pCMV-SCAP, pLVX-SCAP-EGFP, and pCMV-mCherry-KDEL were developed in our laboratory. pCMV-INSIG-2(F115A)-Myc and pLVX-SCAP(D443N)-EGFP were constructed using the QuikChange site-directed kits and confirmed by sequencing. The primers used are listed in Supplemental Table S1.

### Cell culture

Chinese hamster ovary (CHO), HeLa, Huh-7, and human embryonic kidney 293T (HEK293T) cells were grown in monolayer cultures at 37°C in a 5% CO_2_ environment. CHO cells were maintained in medium A, which is a 1:1 mixture of DMEM and Ham's F-12 medium, containing 100 units/ml of penicillin and 100 μg/ml of streptomycin sulfate, supplemented with 5% FBS. HeLa, Huh-7, and HEK293T cells were maintained in medium B (DMEM containing 100 units/ml of penicillin and 100 μg/ml of streptomycin sulfate), supplemented with 10% FBS. The 5% LPDS medium consisted of either medium A or B, supplemented with 5% LPDS and 10 μM mevalonate. The cholesterol depletion medium consisted of either medium A or B, supplemented with 5% LPDS, 1 μM lovastatin, and 10 μM mevalonate.

### Generation of the cell lines

HeLa cells stably expressing pLVX-HMGCR(TM1-8)-EGFP were generated by transfecting the cells with lentivirus expressing pLVX-HMGCR(TM1-8)-EGFP for 48 h. The cells were then switched to medium B supplemented with 10% FBS and selected in the presence of 1 mg/ml of G418 for 2 weeks. Single-cell colonies stably expressing pLVX-HMGCR(TM1-8)-EGFP were confirmed for EGFP expression through immunoblotting and analyzed using the anti-EGFP antibody.

### Immunoblot analysis

Cells were harvested and lysed in RIPA buffer (50 mM Tris-HCl, pH 8.0, 150 mM NaCl, 2 mM MgCl_2_, 1.5% NP-40, 0.1% SDS, and 0.5% sodium deoxycholate) supplemented with protease inhibitors (1 mM PMSF, 0.1% protease cocktail, 5 μM MG132, 5 μg/ml pepstatin A, 25 μg/ml *N*-acetyl-leucyl-leucyl-norleucinal, 10 μg/ml leupeptin, and 250 μM DTT). Protein concentrations were determined using the BCA kit (23225; Thermo Fisher Scientific). Lysates were mixed with 4× loading buffer (150 mM Tris-HCl, pH 6.8, 12% SDS, 30% glycerol, 6% 2-mercaptoethanol, and 0.02% bromophenol blue) and membrane solubilization buffer (62.5 mM Tris-HCl, pH 6.8, 15% SDS, 8 M urea, 10% glycerol, and 100 mM DTT) and then incubated for 30 min at 37°C. Samples were resolved by SDS-PAGE, transferred to PVDF membranes, and blocked in 5% milk in Tris-buffered saline with Tween-20 (25 mM Tris, 137 mM NaCl, 2.7 mM KCl, pH 7.4, and 0.075% Tween-20). Immunoblots were incubated with primary antibodies at 4°C overnight, followed by incubation with secondary antibodies at room temperature for 1 h. After washing three times with Tris-buffered saline with Tween-20, the immunoblots were detected using ECL Western Blotting Substrate (32106; Pierce), and images were captured using digital charge-coupled device imaging (Tanon 5200 Multi or eBlot) or X-ray films.

### Coimmunoprecipitation

To analyze the interaction between INSIG and SCAP, cells were lysed in immunoprecipitation (IP) buffer containing 1% Triton X-100 in PBS, 5 mM EDTA, and 5 mM EGTA, supplemented with 0.2% protease cocktail inhibitors, 10 μM MG-132, and 10 μg/ml leupeptin. The lysate was homogenized by needling 30 times and then centrifuged at 16,000 *g* for 10 min. The resulting supernatant was incubated with 50 μl of anti-c-Myc agarose beads for 16 h at 4°C, washed five times with IP buffer, and eluted with 100 μl of 2× loading buffer at 95°C for 10 min. After centrifugation, 90 μl of supernatant was mixed with 90 μl of membrane solubilization buffer. The samples were then separated by SDS-PAGE and immunoblotted with the specified antibodies.

### Ubiquitination of HMGCR

Cells were transfected and treated as described in the figure legend. Cells were lysed in HMGCR-IP buffer (1× PBS containing 1% [v/v] NP-40, 1% [w/v] deoxycholate, 5 mM EDTA, 5 mM EGTA, 0.1 mM leupeptin, protease inhibitors, 10 μM MG132, and 10 mM *N*-ethylmaleimide). Lysates were immunoprecipitated at 4°C with 50 μl of anti-FLAG agarose beads for 4 h. After incubation, the beads were washed three times with HMGCR-IP buffer at 4°C and then boiled at 95°C for 10 min. Aliquots were subjected to immunoblot analysis. To detect the ubiquitination of endogenous HMGCR, HeLa cells were treated as described in the figure legend. Cells were lysed in HMGCR-IP buffer. Six micrograms of polyclonal antibody against HMGCR were used along with 100 μl of protein A/G agarose beads to precipitate HMGCR. Aliquots were then subjected to immunoblot analysis.

### In vitro ubiquitination of HMGCR

The cell membrane fractions were prepared from sterol-depleted HeLa cells and analyzed by an in vitro ubiquitination assay in a final volume of 0.3 ml of buffer (25 mM HEPES-KOH at pH 7.3, 115 mM potassium acetate, 5 mM sodium acetate, 2.5 mM MgCl2, and 0.5 mM sodium EGTA) containing the ATP-regenerating system (2 mM HEPES-KOH at pH 7.3, 1 mM magnesium acetate, 1 mM ATP, 30 mM creatine phosphate, and 0.05 mg/ml creatine kinase), 0.1 mg/ml FLAG-ubiquitin, 1.6 μg/ml E1 (UBE1), 1.6 μg/ml E2 (UBE2G2, Ubc7), and with or without 25-HC (1 μg/ml) and LY295427 (20 μM) at 37°C for 30 min. The reaction mixture was combined with 300 μl of 1% NP-40 buffer containing protease inhibitors, as previously described, and then passed through a needle 30 times. The solutions were centrifuged at 13,200 rpm for 10 min at 4°C. Six micrograms of polyclonal antibody against HMGCR and 100 μl of protein A/G agarose beads were used to precipitate HMGCR. Aliquots were then subjected to immunoblot analysis.

### Immunostaining

Cells were fixed with 4% paraformaldehyde for 30 min and then permeabilized with 1× PBS containing 0.1% Triton X-100 for 5 min. After permeabilization, cells were blocked with 1% BSA for 30 min, followed by incubation with primary and secondary antibodies diluted in 1% BSA for 1 h at room temperature. Cells were analyzed using a Leica confocal microscope.

### Real-time PCR

Total RNA was extracted from cells using TRIzol reagent (#T9424; Sigma) and reverse transcribed using oligo(dT) primers and M-MLV reverse transcriptase (Promega). The complementary DNAs were then subjected to quantitative real-time PCR using a Bio-Rad CFX384 Real-Time System.

### Photoaffinity-coupled click chemistry labeling

The photoaffinity labeling assay was conducted following a previously described method with minor adjustments. CHO cells were seeded in 10-cm dishes, transfected with plasmids expressing INSIG-1, INSIG-2, and SCAP, and then treated with or without the LY295427 probe, as specified in the figure legends. Cells were harvested and irradiated with UV light at 365 nm for 30 min on ice. Subsequently, the cells were lysed in RIPA buffer containing protease inhibitors and then dialyzed to remove unbound probes. A biotin tag was attached to the probe using click chemistry, which involved sequentially mixing the following reagents at final concentrations: 100 μM biotin-N3, 1 mM TBTA, 1 mM CuSO_4_, and 2.5 mM sodium ascorbate. The reaction took place for 1.5 h at 27°C, followed by dialysis to remove excess biotin-N3. After adding 50 μl of high-capacity Neutravidin beads, the mixture was incubated for 4 h at 4°C. The bound proteins were then eluted using 80 μl of 2× loading buffer and boiled at 95°C for 10 min. The resulting supernatants were mixed with an equal amount of membrane protein solubilization buffer and incubated at 37°C before undergoing SDS-PAGE and immunoblotting.

### Chemical synthesis and characterization

The syntheses and characterizations of LY295427 and the LY295427 probe are described in the Supplemental Methods (20).

## Results

### LY295427 reverses the suppression of SREBP processing mediated by 25-HC

**Fig. 1:**
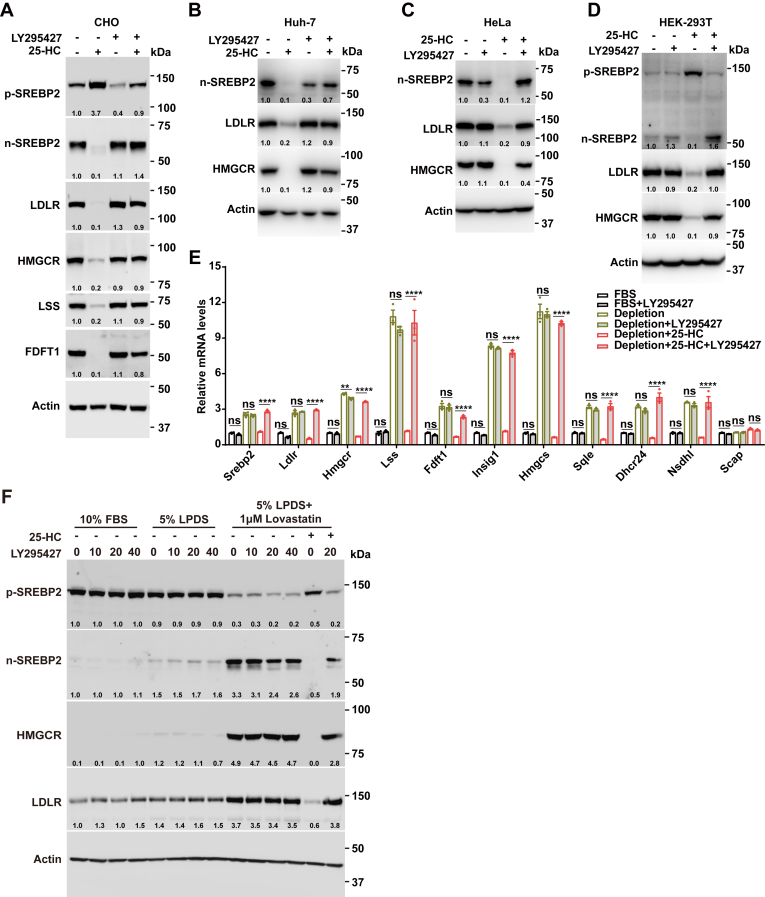
LY295427 reverses the suppression of SREBP processing mediated by 25-HC. A–D: CHO, Huh-7, HeLa, and HEK293T cells were incubated in cholesterol depletion medium (5% LPDS and 1 μM lovastatin) supplemented with or without 1 μg/ml 25-HC and 20 μM LY295427 for 24 h. The cells were harvested and subjected to immunoblotting. E: CHO cells were incubated in medium containing lipoproteins (e.g., 10% FBS) or cholesterol depletion medium supplemented with or without 1 μg/ml 25-HC and 20 μM LY295427 for 24 h. The cells were collected and analyzed by RT-PCR. Data were normalized to control cells incubated in FBS and are presented as mean ± SEM. Statistical significance was determined using a one-way ANOVA. ∗*P* < 0.1; ∗∗*P* < 0.01; ∗∗∗*P* < 0.001; ∗∗∗∗*P* < 0.0001; ns, not significant. F: CHO cells were incubated in 10% FBS, 5% LPDS, or cholesterol depletion medium supplemented with 1 μg/ml 25-HC and different concentrations of LY295427 for 24 h. The cells were harvested and subjected to immunoblotting. The results shown are representative of three independent experiments.

To investigate the effect of the compound LY295427 antagonizing 25-HC, we treated CHO cells with or without 25-HC and LY295427, whose chemical structures are shown in Supplemental Fig. S1. Western blot analysis revealed that 25-HC decreased the protein levels of nuclear SREBP2 (n-SREBP2), LDLR, HMGCR, LSS, and FDFT1 (Fig. 1A). However, this effect was reversed by LY295427. We then tested several different cell lines. In Huh-7, HeLa, and HEK293T cells, LY295427 also inhibited the effects of 25-HC (Fig. 1B–D). Next, we performed quantitative PCR to evaluate the mRNA levels of SREBP2 and its target genes, including *Ldlr*, *Hmgcr*, *Lss*, *Fdft1*, *Insig1*, 3-hydroxy-3-methylglutaryl-CoA synthase (*Hmgcs*), squalene monooxygenase (Sqle), Δ24-dehydrocholesterol reductase (*Dhcr24*), and sterol-4-alpha-carboxylate 3-dehydrogenase (*Nsdhl*). The data indicated that cholesterol depletion (5% LPDS plus 1 μM lovastatin) increased the expression of these genes, and 25-HC downregulated their expression under cholesterol depletion conditions; however, the effect of 25-HC was completely reversed by LY295427 (Fig. 1E). Furthermore, we treated CHO cells cultured in medium containing 10% FBS, 5% LPDS, or cholesterol depletion medium with different concentrations of LY295427 (Fig. 1F). In these conditions, LY295427 did not alter the protein levels of n-SREBP2. SREBP is inhibited by LDL-derived cholesterol under 10% FBS and suppressed by sterol intermediates (e.g., testis meiosis-activating sterol [T-MAS] and follicular fluid meiosis-activating sterol [FF-MAS]) under 5% LPDS (Fig. 1F) (12, 13). The n-SREBP2 has been fully induced in the cholesterol depletion condition. These results were consistent with the previous finding that LY295427 did not reverse the LDL-cholesterol-mediated SREBP inhibition (19). However, LY295427 effectively antagonized the 25-HC-mediated regulation of SREBP2 (Fig. 1A–D and F, the last two lanes).

### LY295427 prevents the 25-HC-induced interaction between SCAP and INSIG-1

**Fig. 2:**
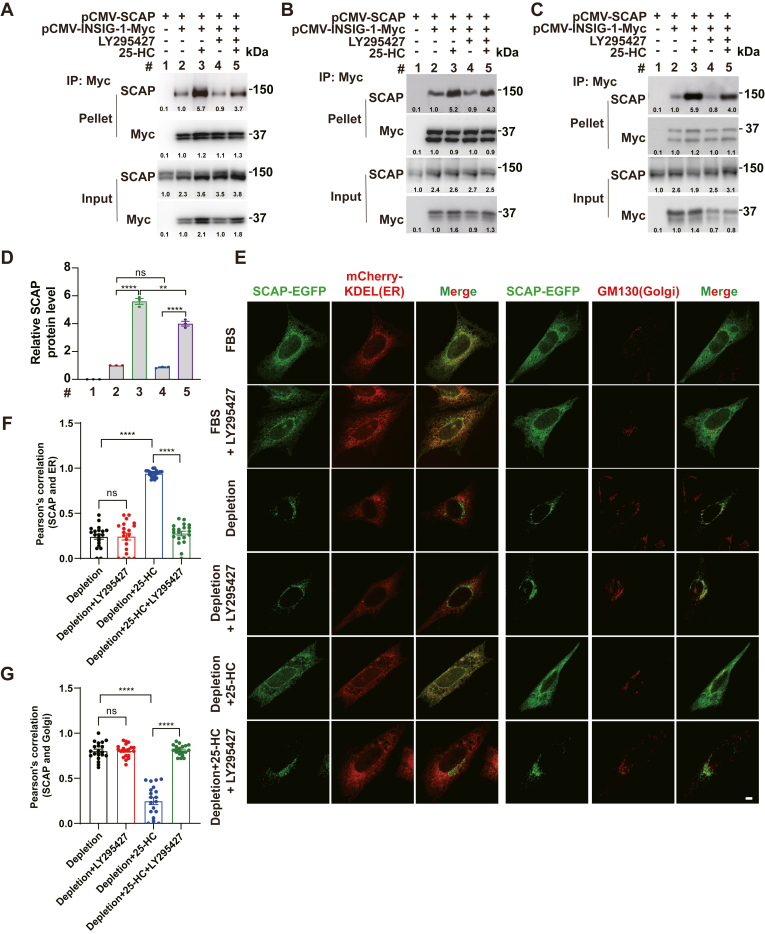
LY295427 prevents the 25-HC-induced interaction between SCAP and INSIG-1. A–C: HEK293T cells were transfected with the indicated plasmids, depleted of sterols, and then treated with or without 1 μg/ml 25-HC and 20 μM LY295427 for 5 h. Lysates were pulled down using anti-Myc antibody-coupled agarose and analyzed by immunoblotting. In the quantification, “1.0” is the normalization baseline. The experiments were repeated three times. D: Quantification of SCAP protein in (A–C). E: CHO cells were transfected with the indicated plasmids and incubated in 10% FBS or cholesterol depletion medium supplemented with or without 1 μg/ml 25-HC and 20 μM LY295427 for 24 h. The cells were fixed and stained with the Golgi marker anti-GM130 antibody. The scale bar represents 5 μm. F and G: Quantified the colocalization of ER, Golgi apparatus, and SCAP-EGFP fluorescence signals in B. The data are presented as means ± SEM with n = 20 cells for each group (from left to right). Statistical significance was determined using a one-way ANOVA. The numerical data supporting this study are available as source data. ∗*P* < 0.1; ∗∗*P* < 0.01; ∗∗∗*P* < 0.001; ∗∗∗∗*P* < 0.0001; ns, not significant.

To investigate whether LY295427 influences 25-HC-induced interaction between INSIG and SCAP, we cotransfected cells with INSIG-1 and SCAP expression plasmids. The co-IP results revealed that 25-HC increased the interaction between SCAP and INSIG-1. In contrast, LY295427 partially alleviates the 25-HC-induced interaction between SCAP and INSIG-1 (Fig. 2A–D). Confocal microscopy studies indicated that SCAP was translocated from the ER to the Golgi under cholesterol depletion conditions. 25-HC completely prevented the transport of SCAP from the ER to the Golgi, causing SCAP to stay in the ER. However, SCAP was transported to the Golgi when the cells were treated with both 25-HC and LY295427 (Fig. 2E). The colocalization of ER, Golgi apparatus, and SCAP-EGFP fluorescence signals was quantified (Fig. 2F and G). Thus, LY295427 prevents 25-HC-induced interaction between SCAP and INSIG-1 and therefore promotes the ER-to-Golgi translocation of SCAP.

### LY295427 reverses the inhibition of SREBP processing by 25-HC, 25-hydroxylanosterol, and cholesterol

**Fig. 3:**
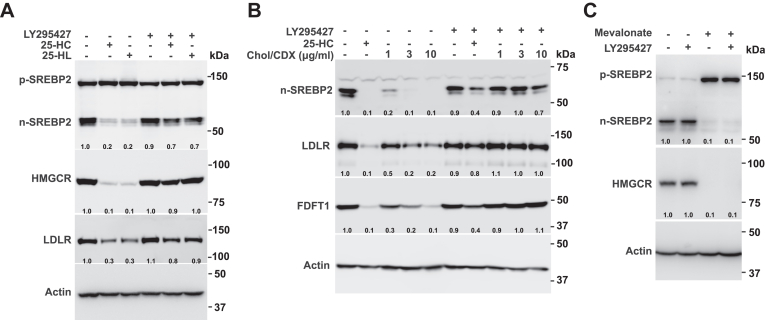
LY295427 reverses the inhibition of SREBP processing by 25-HC, 25-HL, and cholesterol but not by mevalonate and LDL. A: CHO cells were incubated in cholesterol depletion medium supplemented with or without 1 μg/ml 25-HC, 1 μg/ml 25-HL, and 20 μM LY295427 for 24 h. The cells were harvested and subjected to immunoblotting. B: HeLa cells were incubated in cholesterol depletion medium supplemented with or without 1 μg/ml 25-HC, different concentrations of Chol/CDX, and 20 μM LY295427 for 24 h. The cells were harvested and subjected to immunoblotting. C: HeLa cells were incubated in cholesterol depletion medium supplemented with or without 10 mM mevalonate and 20 μM LY295427 for 12 h. The cells were harvested and subjected to immunoblotting.

Besides 25-HC, cholesterol in methyl-β-cyclodextrin (Chol/CDX) and 25-hydroxylanosterol (25-HL) (21) (Supplemental Fig. S1C) can also inhibit SREBP processing. We then tested whether LY295427 antagonized 25-HL and Chol/CDX. The results showed that 25-HC, 25-HL, and Chol/CDX inhibited SREBP2 processing as evidenced by the decrease in n-SREBP2. As a consequence, the protein levels of HMGCR, LDLR, and FDFT1 were also reduced since they were transcriptionally regulated by n-SREBP2. However, this decrease was reversed by LY295427 (Fig. 3A and B). Thus, LY295427 also acted as an antagonist against 25-HL and cholesterol/CDX. Mevalonate suppressed SREBP2 expression since sterol intermediates (e.g., T-MAS and FF-MAS) can inhibit SREBP processing (12, 13). However, this n-SREBP2 decrease was not reversed by LY295427. So, LY295427 was unable to antagonize the effects of mevalonate (Fig. 3C).

### LY295427 suppresses the ubiquitination of HMGCR mediated by 25-HC

**Fig. 4:**
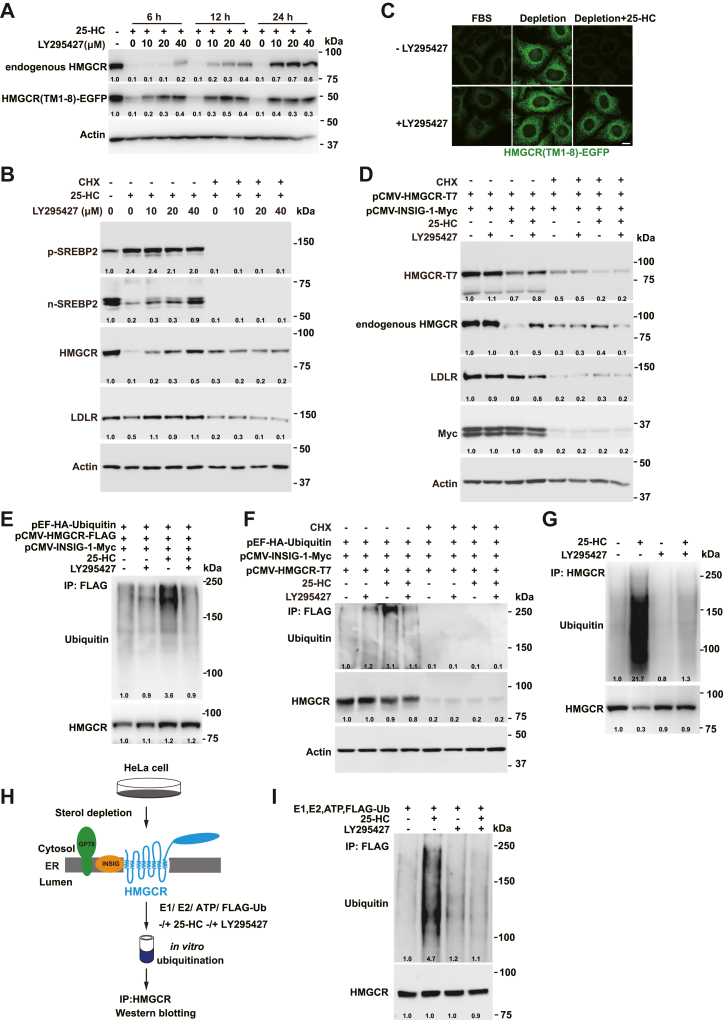
LY295427 suppresses the ubiquitination of HMGCR induced by 25-HC. A: HeLa cells stably expressing HMGCR (TM1-8)-EGFP were incubated in cholesterol depletion medium supplemented with or without 1 μg/ml 25-HC and LY295427 for different hours. The cells were harvested and subjected to immunoblotting. Lysates were immunoblotted with antiendogenous HMGCR (A9), anti-EGFP, and anti-Actin antibodies. B: CHO cells were incubated in cholesterol depletion medium supplemented with or without 10 μg/ml CHX, 1 μg/ml 25-HC, and LY295427 for 5 h. The cells were harvested and subjected to immunoblotting. C: HeLa cells stably expressing HMGCR (TM1-8)-EGFP were incubated in 10% FBS or cholesterol depletion medium supplemented with or without 1 μg/ml 25-HC and 20 μM LY295427 for 24 h. The cells were harvested and subjected to immunofluorescence. The scale bar represents 5 μm. D: CHO cells were transfected with the indicated plasmids, depleted of sterols, and then treated with or without 10 μg/ml CHX, 1 μg/ml 25-HC, and 20 μM LY295427 for 5 h. The cells were analyzed by immunoblotting. E: CHO cells were transfected with the indicated plasmids, depleted of sterols, and treated with 10 μM MG132 supplemented with or without 1 μg/ml 25-HC and 20 μM LY295427 for 1 h. Lysates were immunoprecipitated using anti-FLAG beads. Input and pellet fractions were immunoblotted with anti-FLAG and hemagglutinin (HA) antibodies. F: CHO cells were transfected with the indicated plasmids, depleted of sterols, and treated with or without 10 μg/ml CHX, 1 μg/ml 25-HC, and 20 μM LY295427 for 1 h. Lysates were immunoprecipitated using anti-FLAG beads. Input and pellet fractions were immunoblotted with anti-FLAG, anti-Actin, and HA antibodies. G: HeLa cells were depleted of sterols and then treated with or without 25-HC and LY295427 for 1 h. The cells were harvested, and lysates were immunoprecipitated using protein A/G beads coupled with the anti-HMGCR antibody. Input and pellet fractions were immunoblotted with anti-HMGCR and antiubiquitin antibodies. H and I: A schematic representation of the experimental design for the in vitro ubiquitination of the HMGCR assay is shown, as described in the Materials and methods section. The results presented are representative of three independent experiments.

It is known that 25-HC not only inhibits SREBP processing but also induces the ubiquitination and proteasomal degradation of HMGCR (1). To investigate whether LY295427 antagonizes 25-HC-induced HMGCR degradation, we treated HeLa cells stably expressing HMGCR (TM-1-8)-EGFP with or without 25-HC and LY295427. Western blot analysis revealed that 25-HC suppressed the protein levels of both endogenous HMGCR and HMGCR (TM-1-8)-EGFP. However, this effect was reversed by LY295427 in concentration- and time-dependent manners (Fig. 4A). When CHX was used to block protein synthesis, the antagonistic effect of LY295427 on 25-HC was abolished (Fig. 4B). Consistently, fluorescence-activated cell sorting and confocal microscopy revealed that 25-HC decreased the fluorescence of HMGCR (TM-1-8)-EGFP, but this effect was reversed by LY295427 (Supplemental Fig. S1D–H and Fig. 4C). Next, we cotransfected cells with HMGCR and INSIG-1 expression plasmids. Western blot analysis indicated that 25-HC decreased the protein level of HMGCR, an effect that was reversed by LY295427. However, in the presence of CHX, the effect of LY295427 is eliminated (Fig. 4D). This result excludes the transcriptional regulation by SREBP and suggests that LY295427 antagonizes the degradation of HMGCR. We also analyzed the ubiquitination of HMGCR in cells. 25-HC increased the ubiquitination of both transfected and endogenous HMGCR, an effect that was blunted by LY295427 (Fig. 4E–G). In the presence of CHX, the effect of LY295427 disappeared (Fig. 4F). We further analyzed the ubiquitination of HMGCR in vitro. The membrane fractions containing HMGCR, INSIG, and the E3 gp78 were prepared from sterol-depleted HeLa cells. The E1, FLAG-ubiquitin, ATP, 25-HC, and LY295427 were added as indicated. After incubation for 30 min, HMGCR was immunoprecipitated, and its ubiquitination was measured (Fig. 4H). As shown in Figure 4I, 25-HC stimulated HMGCR ubiquitination in vitro, and LY295427 blocked this effect. These results suggest that LY295427 antagonizes the 25-HC-induced ubiquitination and degradation of HMGCR in cells and in vitro.

### LY295427 binds to INSIG

**Fig. 5:**
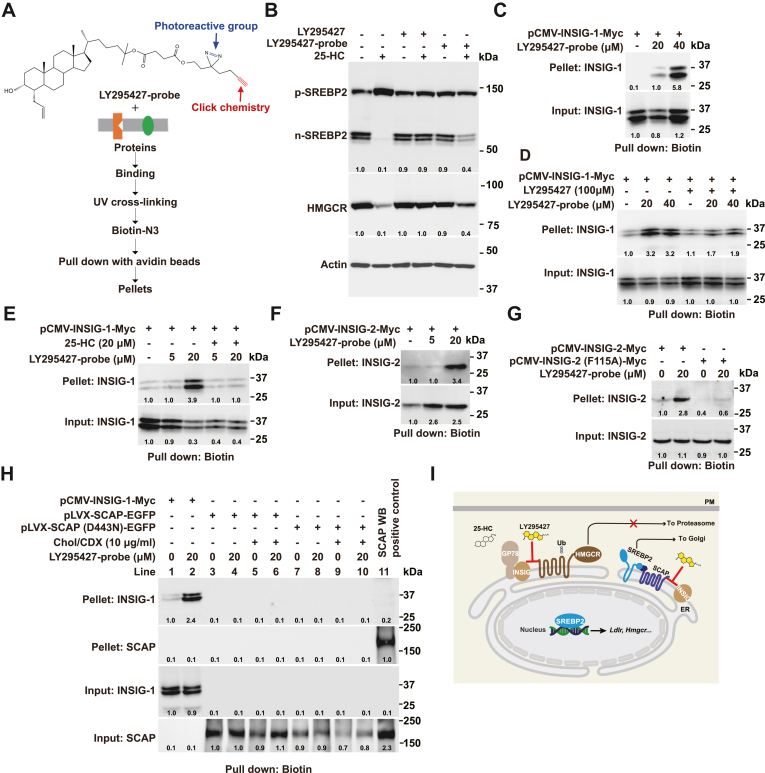
LY295427 binds to INSIG. A: The structure of the LY295427 probe and a schematic illustration of the detection procedure for LY295427-binding proteins. B: CHO cells were incubated in cholesterol depletion medium supplemented with or without 20 μM LY295427, 20 μM LY295427 probe, and 1 μg/ml 25-HC for 24 h. The cells were harvested and subjected to immunoblotting. C–G: CHO cells were transfected with pCMV-INSIG-1-Myc, pCMV-INSIG-2-Myc, or pCMV-INSIG-2 (F115A)-Myc plasmid for 24 h and incubated in cholesterol depletion medium supplemented with or without LY295427 probe, LY295427, and 25-HC for 24 h. The cells were harvested and treated as described in A. Input and pellet were immunoblotted with anti-Myc antibody. H: HEK293T cells were transfected with pLVX-SCAP-EGFP or pLVX-SCAP(D443N)-EGFP plasmid for 24 h and incubated in cholesterol depletion medium supplemented with or without LY295427 probe and cholesterol/CDX for 24 h. The cells were harvested and treated as described in A. Input and pellet were immunoblotted with anti-Myc and anti-SCAP (9D5) antibodies. I: The model diagram illustrating the action of 25-HC and LY295427.

Since 25-HC binds INSIG to regulate SREBP processing, we then hypothesize that LY295427 acts by binding to INSIG-1. To test it, a probe derived from LY295427 was synthesized and named the LY295427 probe. This probe contains the photoreactive group that covalently links to the binding protein under UV reaction and the alkynyl group that can be coupled to azide-biotin via click chemistry. The LY295427 probe was incubated with cholesterol-depleted CHO cells before being exposed to UV light to activate the crosslinking. Following UV irradiation, a biotin tag was attached using click chemistry. The cell lysates were then incubated with avidin beads to pull down LY295427-binding proteins (Fig. 5A). First, we found that the LY295427 probe could antagonize 25-HC-inhibited SREBP processing, although it was less potent than LY295427 in CHO cells (Fig. 5B). Following the procedure shown in Figure 5A, INSIG-1 was precipitated in the presence of the LY295427 probe (Fig. 5C), and this binding could be completed by LY295427 and 25-HC (Fig. 5D and E). INSIG-2 was also precipitated when the LY295427 probe was present (Fig. 5F). The F115A mutation disrupts 25-HC binding to INSIG-2 (22). INSIG-2 (F115A) did not crosslink to the LY295427 probe (Fig. 5G), suggesting that F115 might be involved in 25-HC or LY295427 binding. SCAP (D443N) was resistant to sterol regulation (23). Both WT SCAP and SCAP (D443N) could not link to the LY295427 probe (Fig. 5H). Our results suggest that LY295427 antagonizes 25-HC to regulate SREBP cleavage or HMGCR degradation through competing 25-HC-INSIG interaction.

## Discussion

It has been known that LY295427 can reverse suppression of SREBP processing mediated by oxysterols, including 25-HC. This study investigates the mechanism of LY295427 and finds that LY295427 prevents 25-HC-induced interaction between SCAP and INSIG as well as ER retention of SCAP (Fig. 5I). Using the chemical probe, our results show that LY295427 binds INSIG, which can be completed by 25-HC.

In addition to sterol-regulated processing of SREBP, the sterol-induced degradation of HMGCR is another essential feedback regulatory mechanism governing cholesterol biosynthesis. INSIG is a key player in both processes. INSIG brings the associated E3, including gp78, TRC8, or RNF145, to HMGCR in the presence of oxysterols or sterol intermediates. In this study, we find that LY295427 also prevents the 25-HC-induced ubiquitination and degradation of HMGCR. Notably, LY295427 blocks the ubiquitination of HMGCR in vitro induced by 25-HC. The in vitro system only has the membrane fraction, E1, ATP, and FLAG-ubiquitin without cytosol or intact cell structure. So, these results further support the idea that LY295427 acts by binding to INSIG.

A previous study demonstrated that LY295427 overcame the suppression of SREBP processing induced by 25-HC but not by LDL-derived cholesterol (14). However, we further show that LY295427 can override the effect of cholesterol/CDX (Fig. 3B). It is known that cholesterol binds to loop 1 of SCAP but not INSIG. If LY295427 is capable of competing with cholesterol for binding to SCAP, why does it fail to overcome the suppression effect of LDL-derived cholesterol? One possible mechanism is that LDL might deliver a large amount of cholesterol to the ER, a level that cannot be overridden by LY295427. Additionally, LY295427 may affect SCAP or the SREBP pathway through mechanisms other than direct competition with cholesterol, such as altering SCAP conformation or interacting with other regulatory components. Further studies are needed to explore how LY295427 functions under these conditions and why it cannot fully counteract the effect of LDL-derived cholesterol.

Our findings showed that under sterol-depletion conditions plus 25-HC, LY295427 significantly increased the transcription of *Insig1* compared to treatment with 25-HC alone (Fig. 1E). This condition was consistent with that used in a previous study, in which the author compared sterol depletion plus 25-HC to sterol depletion plus 25-HC and LY295427 (24). However, LY295427 did not increase *Insig-1* expression in 10% FBS or cholesterol depletion conditions (Fig. 1E). The reasons are that LY295427 does not antagonize LDL-cholesterol in 10% FBS, and SREBP has been highly activated in cholesterol depletion conditions. Meanwhile, our data demonstrated that INSIG-2, but not INSIG-2 (F115A), was crosslinked to the LY295427 probe (Fig. 5G), indicating that LY295427 binding to INSIG-2 depends on the F115 residue (22). These findings support the notion that LY295427 competitively antagonizes the inhibitory effect of 25-HC on SREBP by targeting the F115-dependent site on INSIG-2. We were unable to functionally validate that all effects of LY295427 absolutely depend on its binding to INSIG by reconstituting INSIG-deficient cells with WT and LY295427-binding-deficient (e.g., INSIG2-F115A) INSIG. Such an experiment would provide the most direct genetic evidence for our competition model. This constitutes an important goal for future research. While our data do not show stabilization of the SCAP-INSIG complex by LY295427 (Fig. 2A), we propose that it might still influence the complex through this interaction. Although LY295427 reduces the SCAP–INSIG interaction in the context of 25-HC, the magnitude of change is modest and largely correlates with INSIG abundance rather than altered binding affinity. However, further studies, such as cryo-EM or binding assays, are needed to validate this hypothesis and confirm LY295427’s binding to this site.

SREBP is inhibited by LDL-derived cholesterol under a medium containing lipoproteins (e.g., 10% FBS) and is suppressed by sterol intermediates (e.g., T-MAS and FF-MAS) under 5% LPDS (Fig. 1F) (12, 13). Unexpectedly, LY295427 did not reverse SREBP inhibition under medium containing lipoproteins (e.g., 10% FBS) or 5% LPDS conditions but markedly reversed 25-HC-mediated SREBP inhibition (Fig. 1F). This result is consistent with previous findings showing that LY295427 fails to reverse LDL-cholesterol-mediated SREBP inhibition (19). We also observed that LY295427 counteracted cholesterol/CDX-induced SREBP inhibition (Fig. 3). Collectively, these data suggest that the mechanisms underlying SREBP inhibition by 25-HC and cholesterol/CDX differ from those of LDL-cholesterol and sterol intermediates. This may be attributed to 25-HC and cholesterol delivered by CDX binding to SCAP-INSIG at sites distinct from those targeted by LDL-cholesterol and sterol intermediates. Furthermore, 25-HC is known to regulate intracellular cholesterol trafficking. Therefore, we cannot fully exclude the possibility that LY295427 acts indirectly by influencing cholesterol transport between compartments, such as the plasma membrane, ER, and lysosomes. Future studies utilizing tools, such as NPC1- or ACAT1-deficient cells, will help elucidate whether LY295427 also affects these processes.

In the presence of 25-HC, LY295427 increased HMGCR(TM1-8)-EGFP level (Fig. 4A and B). It had no obvious effect in a medium containing lipoproteins (e.g., 10% FBS) or a cholesterol depletion condition (Fig. 4C). The reasons might be that LY295427 does not antagonize LDL-cholesterol, and HMGCR(TM1-8)-EGFP had been highly induced in a cholesterol depletion condition. Treatment with CHX suppresses SREBP, HMGCR, and INSIG expression. Under this condition, LY295427 fails to induce any increase in n-SREBP-2 and HMGCR. The ubiquitination of HMGCR is abolished too (Fig. 4B, D and F).

In summary, we here show that LY295427 can reverse the regulatory effects of 25-HC on SREBP processing and HMGCR degradation through binding to INSIG.

## Data Availability

All data used for the research are described in the article. Data will be available upon request from the corresponding author.

## Supplemental Data

This article contains supplemental data (20).

## Conflict of interest

The authors declare that they have no conflicts of interest with the contents of this article.
