# Supplementary material for: The compound LY295427 antagonizes 25-hydroxycholesterol through binding to INSIG
Source: J Lipid Res. 2026 Mar 6;67(4):101015. doi: 10.1016/j.jlr.2026.101015 (PMC13090600; doi:10.1016/j.jlr.2026.101015)

**Title: The compound LY295427 antagonizes 25-hydroxycholesterol** **through binding to INSIG**


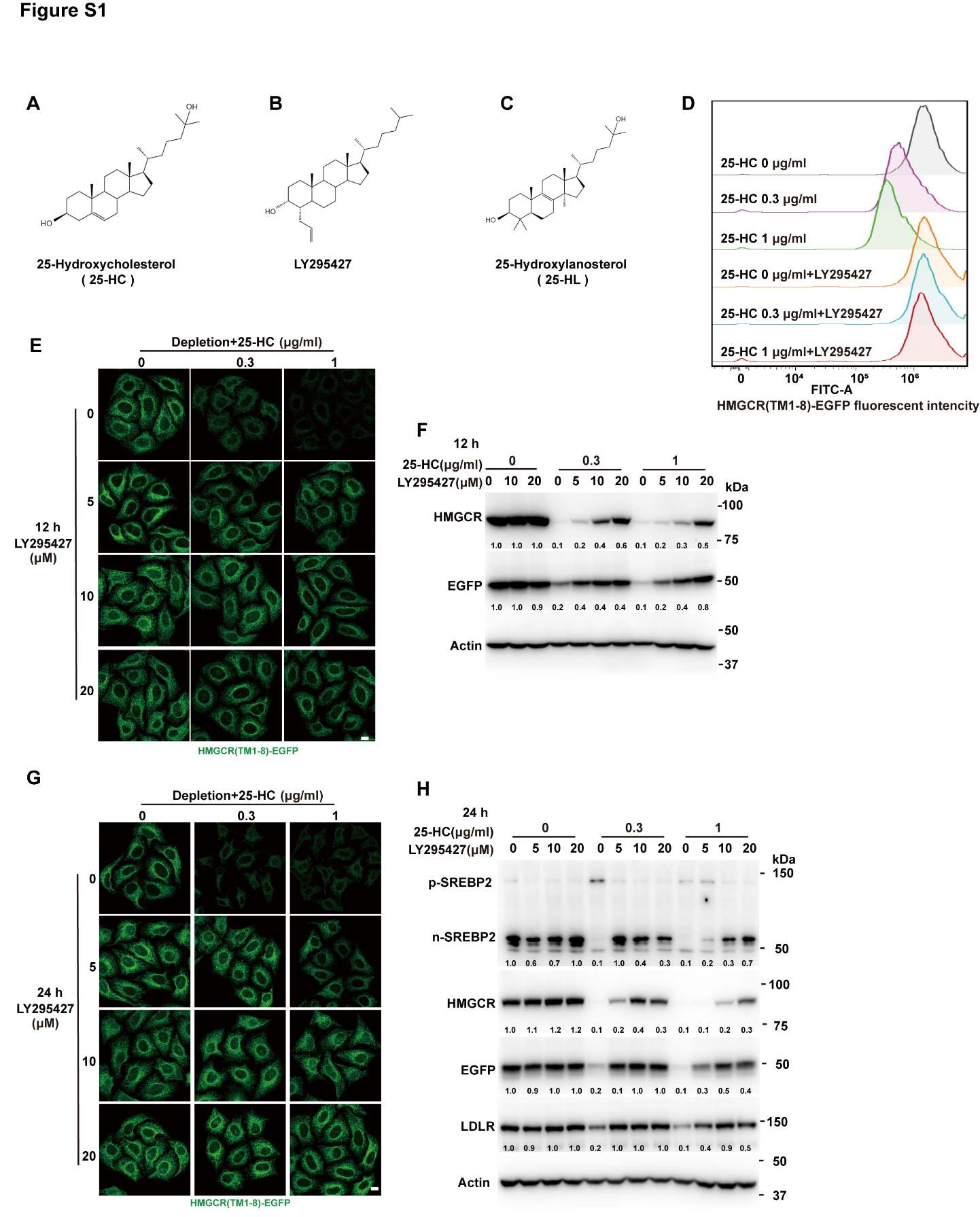
**Supplemental Information**

**Figure S1. LY295427 reverses the suppression of HMGCR mediated by 25-HC.**

A-C, Chemical structures of 25-HC, LY295427 and 25-HL. D, HeLa cells stably expressing HMGCR(TM1-8)-EGFP were incubated in indicated medium for 24 hours. Cells were harvested and subjected to fluorescence-activated cell sorting. E-H, HeLa cells stably expressing HMGCR(TM1-8)-EGFP were incubated in cholesterol-depletion medium with or without varying concentrations of 25-HC and LY295427 for 12 or 24 hours, followed by immunofluorescence and immunoblotting analysis. Results shown are representative of three independent experiments.

**Chemical synthesis and characterization.**

*General*

All reagents and chemicals were purchased from commercial suppliers and used without further purification unless otherwise stated. When needed, the reactions were carried out in oven-dried glassware under a positive pressure of dry N_2_. Column chromatography was performed on silica gel (QingDao, 200-300 mesh) using the indicated eluents. Thin-layer chromatography was carried out on silica gel plates (QingDao) with a layer thickness of 0.25mm. ^1^H (500 and 600 MHz) and ^13^C (125 and 150 MHz) NMR spectra were recorded on Bruker AM 500 and 600 spectrometers with CDCl_3_ as solvent and tetramethylsilane (TMS) as the internal standard. All chemical shift values were reported in units of *δ* (ppm). The following abbreviations were used to indicate the peak multiplicity: *s* = singlet, *d* = doublet, *t* = triplet, *m* = multiplet, *dd* = doublet/ doublet. High-resolution mass data were obtained on a BrukermicroOTOF-Q II spectrometer.

**Scheme 1.** **Synthesis of LY295427.**^1^

Reagents and conditions: (a) (i) Li, NH_3_, *t*-BuOH, tetrahydrofuran, -78 ℃, 15 min; (ii) allyl iodide, -78 ℃, 2.5h (31%); (b) K-selectride, tetrahydrofuran, rt, 1h (71%).

*(4S,5S,8S,9S,10R,13R,14S,17R)-4-allyl-10,13-dimethyl-17-((R)-6-methylheptan-2-yl)hexadecahydro-3H-cyclopenta[a]phenanthren-3-one (****1****).* To a solution of liquid ammonia (60 mL) was added lithium chips (54.2 mg, 7.8 mmol) at -78 ℃, and the mixture was stirred for 30 minutes under a N_2_ atmosphere, affording a lithium ammonia solution. Cholest-4-en-3-one (1.0 g, 2.6 mmol) in anhydrous *t*-BuOH (0.24 mL) and tetrahydrofuran (40 mL) was added dropwise to this lithium ammonia solution. The reaction mixture was stirred for 15 min, then 1,3-piperlene (1 mL) was added to quench the excess of lithium. Allyl iodide (1.3 g, 7.8 mmol) was added to the reaction mixture and stirred for 2.5 h at -78 ℃ under a N_2_ atmosphere. Then, the reaction mixture was quenched with an aqueous solution of NH_4_Cl (30 mL) and extracted with AcOEt (20 mL × 3). The organic layer was washed with brine, dried over anhydrous Na_2_SO_4_, and concentrated. The residue was purified by silica gel chromatography (petroleum ether/AcOEt, 80/1, v/v) to give **1** (340 mg, 31%) as a white solid. ^1^H NMR (500 MHz, CDCl_3_) *δ* 5.81–5.71 (m, 1H), 5.05–4.91 (m, 2H), 2.49–2.36 (m, 2H), 2.34–2.19 (m, 3H), 2.03–1.94 (m, 2H), 1.86–1.77 (m, 1H), 1.75–1.67 (m, 2H), 1.59–1.46 (m, 3H), 1.41–1.29 (m, 7H), 1.29–1.17 (m, 2H), 1.16–1.06 (m, 6H), 1.05 (s, 3H), 1.01–0.93 (m, 2H), 0.89 (d, *J* = 6.5 Hz, 3H), 0.86 (dd, *J* = 6.6, 2.3 Hz, 6H), 0.83–0.76 (m, 1H), 0.74–0.67 (m, 1H), 0.67 (s, 3H). ^13^C NMR (125 MHz, CDCl_3_) *δ* 212.53, 136.58, 116.00, 56.32, 56.27, 54.10, 50.22, 49.87, 42.54, 39.96, 39.51, 38.79, 38.22, 36.25, 36.16, 35.80, 34.91, 31.79, 29.84, 28.28, 28.02, 25.35, 24.18, 23.85, 22.83, 22.56, 21.39, 18.66, 12.78, 12.07.

*(3R,4S,5S,8S,9S,10R,13R,14S,17R)-4-allyl-10,13-dimethyl-17-((R)-6-methylheptan-2-yl)hexadecahydro-1H-cyclopenta[a]phenanthren-3-ol (****LY295427****).* To a solution of compound **1** (326 mg, 0.76 mmol) in anhydrous tetrahydrofuran (15 mL) was added K-selectride (1.2 mL, 1.2 mmol) at -78 ℃. The reaction mixture was stirred for 1 h at room temperature under a N_2_ atmosphere. Then, the mixture was poured into water and extracted with AcOEt (10 mL × 3). The organic layer was washed with brine, dried over anhydrous Na_2_SO_4_, and concentrated. The residue was purified by silica gel chromatography (petroleum ether/AcOEt, 60/1, v/v) to give **LY295427** (234 mg, 71%) as a white solid. ^1^H NMR (500 MHz, CDCl_3_) *δ* 5.94–5.79 (m, 1H), 5.06 (dd, *J* = 37.2, 13.5 Hz, 2H), 3.90 (d, *J* = 2.8 Hz, 1H), 2.31–2.23 (m, 1H), 2.02–1.92 (m, 2H), 1.85–1.76 (m, 1H), 1.74–1.65 (m, 3H), 1.61–1.52 (m, 4H), 1.50–1.44 (m, 3H), 1.38–1.19 (m, 9H), 1.15–1.06 (m, 4H), 1.05–0.94 (m, 4H), 0.90 (d, *J* = 6.5 Hz, 3H), 0.86 (dd, *J* = 6.6, 2.4 Hz, 6H), 0.81 (s, 3H), 0.78–0.71 (m, 1H), 0.65 (s, 3H). ^13^C NMR (125 MHz, CDCl_3_) *δ* 138.37, 115.81, 67.88, 56.59, 56.21, 54.46, 43.56, 42.46, 40.25, 40.09, 39.53, 36.52, 36.18, 35.81, 34.91, 34.39, 32.23, 32.13, 29.01, 28.27, 28.02, 24.14, 23.85, 23.83, 22.83, 22.57, 20.98, 18.66, 12.36, 12.07.

**Scheme 2. Synthesis of LY295427-probe**.

Reagents and conditions: (a) iodine, triphenylphosphine, imidazole, dichloromethane, rt, 2 h (98%); (b) methyl acrylate, nickel chloride hexahydrate, zinc, pyridine, rt, 3 h (85%); (c) ethylene glycol, triethoxy methane, *p*-toluenesulfonic acid, 40 ℃, 40 min; (d) methylmagnesium bromide, tetrahydrofuran, rt, 1h; (e) 2N HCl, tetrahydrofuran, rt, 4h (67% over three steps); (f) (i) Li, ammonia, *t*-BuOH, tetrahydrofuran, -78 ℃; (ii) allyl iodide, -78 ℃, 2.5h (75%); (g) succinic anhydride, DMAP, pyridine, reflux, 72 h (67%); (h) K-selectride, tetrahydrofuran, rt, 1h (87%); (i) alkyne-diazirine-OH, EDCI, DMAP, dichloromethane, rt, 4 h (61%).

*(8S,9S,10R,13S,14S,17R)-17-((S)-1-iodopropan-2-yl)-10,13-dimethyl-1,2,6,7,8,9,10,11,12,13,14,15,16,17-tetradecahydro-3H-cyclopenta[a]phenanthren-3-one (****2****).* To a solution of triphenylphosphine (23.8 g, 90.9 mmol) and imidazole (12.4 g, 181.8 mmol) in dichloromethane (100 mL) was added iodine (11.5 g, 90.9 mmol) at 0 ℃ and stirred for 0.5 h. Bisnoralcohol (10 g, 30.3 mmol) was added. The reaction mixture stirred for 2 h at room temperature, then quenched with an aqueous solution of Na_2_S_2_O_3_ and extracted with dichloromethane (80 mL × 3). The organic layer was washed with brine, dried over anhydrous Na_2_SO_4_, and concentrated. The residue was purified by silica gel chromatography (petroleum ether/AcOEt, 10/1, v/v) to give **2** (13 g, 98%) as a white solid. ^1^H NMR (500 MHz, CDCl_3_) *δ* 5.72 (s, 1H), 3.32 (d, *J* = 9.5 Hz, 1H), 3.16 (dd, *J* = 9.6, 4.3 Hz, 1H), 2.45–2.24 (m, 4H), 2.04–1.94 (m, 2H), 1.89–1.81 (m, 2H), 1.73–1.61 (m, 2H), 1.56–1.51 (m, 2H), 1.50–1.36 (m, 1H), 1.28–1.21 (m, 2H), 1.17 (s, 5H), 1.15–1.03 (m, 3H), 1.02 (d, *J* = 5.5 Hz, 3H), 0.95–0.90 (m, 1H), 0.74 (s, 3H). ^13^C NMR (125 MHz, CDCl_3_) *δ* 199.58, 171.37, 123.85, 55.54, 55.32, 53.65, 42.40, 39.29, 38.55, 36.88, 35.69, 35.61, 33.98, 32.88, 31.94, 27.53, 24.04, 21.05, 20.97, 20.73, 17.38, 12.74.

*Methyl(R)-5-((8S,9S,10R,13R,14S,17R)-10,13-dimethyl-3-oxo-2,3,6,7,8,9,10,11,12,13,14,15,16,17-tetradecahydro-1H-cyclopenta[a]phenanthren-17-yl)hexanoate (****3****).* To a solution of nickel chloride hexahydrate (7.0 g, 29.5 mmol) and methyl acrylate (11.4 g, 132.8 mmol) in pyridine (30 mL) was added zinc (8.7 g, 132.8 mmol) at room temperature. The reaction mixture was stirred for 0.5 h at 65 ℃ under a N_2_ atmosphere. After cooling, compound **2** (13.0 g, 29.5 mmol) in pyridine (30 mL) was added and the reaction mixture stirred for 3 h at room temperature. Then, the reaction mixture was filtered through Celite, washing with AcOEt (100 mL). The filtrate was washed with brine, dried over anhydrous Na_2_SO_4_, and concentrated. The residue was purified by silica gel chromatography (petroleum ether/AcOEt, 10/1, v/v) to give **3** (10 g, 85%) as a white solid. ^1^H NMR (500 MHz, CDCl_3_) *δ* 5.72 (s, 1H), 3.66 (s, 3H), 2.46–2.20 (m, 6H), 2.04–1.99 (m, 2H), 1.88–1.80 (m, 2H), 1.74–1.64 (m, 2H), 1.64–1.58 (m, 1H), 1.54–1.45 (m, 3H), 1.45–1.33 (m, 3H), 1.29–1.22 (m, 2H), 1.18 (s, 3H), 1.16–0.95 (m, 6H), 0.93 (d, *J* = 6.6 Hz, 3H), 0.70 (s, 3H).

*(8S,9S,10R,13R,14S,17R)-17-((R)-6-hydroxy-6-methylheptan-2-yl)-10,13-dimethyl-1,2,6,7,8,9,10,11,12,13,14,15,16,17-tetradecahydro-3H-cyclopenta[a]phenanthren-3-one (****6****).* To a solution of compound **3** (7.2 g, 18 mmol) in triethoxy methane (20 mL) and ethylene glycol (10 mL) was added *p*-toluenesulfonic acid (619 mg, 3.6 mmol) at room temperature. The reaction mixture was stirred for 40 min at 40 ℃ under a N_2_ atmosphere. After cooling, the reaction mixture was quenched with an aqueous solution of NaHCO_3_ and extracted with AcOEt (30 mL × 3). The organic layer was washed with brine, dried over anhydrous Na_2_SO_4_, and concentrated to give a crude product **4** as a yellow oil, which was used in the next step without further purification.

To a solution of compound **4** in anhydrous tetrahydrofuran (50 mL) was added a THF solution of methylmagnesium bromide (30 mL, 90 mmol) at 0 ℃. The reaction mixture was stirred for 1 h at room temperature under a N_2_ atmosphere. Then, the reaction mixture was quenched with an aqueous solution of 2N HCl and extracted with AcOEt (30 mL × 3). The organic layer was washed with brine, dried over anhydrous Na_2_SO_4_, and concentrated to give a crude product **5** as a yellow oil, which was used in the next step without further purification.

To a solution of compound **5** in tetrahydrofuran (50 mL) was added an aqueous solution of 2N HCl (30 mL) at room temperature. The reaction mixture was stirred for 4 h at room temperature and concentrated. The residue was purified by silica gel chromatography (petroleum ether/AcOEt, 5/1, v/v) to give **6** (4.8 g, 67% over three steps) as a white solid. ^1^H NMR (600 MHz, CDCl_3_) *δ* 5.70 (s, 1H), 2.45–2.28 (m, 3H), 2.26–2.23 (m, 1H), 2.04–1.97 (m, 2H), 1.85–1.78 (m, 2H), 1.70–1.65 (m, 1H), 1.61–1.56 (m, 1H), 1.54–1.47 (m, 2H), 1.46–1.31 (m, 8H), 1.27–1.22 (m, 2H), 1.20 (s, 6H), 1.16 (s, 3H), 1.13–1.06 (m, 2H), 1.04–0.96 (m, 3H), 0.91 (d, *J* = 6.6 Hz, 3H), 0.69 (s, 3H). ^13^C NMR (150 MHz, CDCl_3_) *δ* 199.67, 171.71, 123.74, 71.04, 56.04, 55.87, 53.80, 44.39, 42.40, 39.62, 38.60, 36.38, 35.71, 35.68, 35.61, 33.98, 32.94, 32.04, 29.36, 29.22, 28.19, 24.17, 21.02, 20.77, 18.61, 17.38, 11.96.

*(4S,5S,8S,9S,10R,13R,14S,17R)-4-allyl-17-((R)-6-hydroxy-6-methylheptan-2-yl)-10,13-dimethylhexadecahydro-3H-cyclopenta[a]phenanthren-3-one* *(****7****)*. By a similar procedure described for compound **1**, compound **7** was obtained as a white solid (yield 75%). ^1^H NMR (500 MHz, CDCl_3_) *δ* 5.81–5.70 (m, 1H), 5.03–4.92 (m, 2H), 2.49–2.35 (m, 2H), 2.34–2.19 (m, 3H), 2.03–1.94 (m, 2H), 1.85–1.75 (m, 1H), 1.75–1.66 (m, 2H), 1.58–1.53 (m, 2H), 1.46–1.26 (m, 11H), 1.25–1.22 (m, 1H), 1.20 (s, 6H), 1.16–1.06 (m, 3H), 1.04 (s, 3H), 1.02–0.94 (m, 2H), 0.90 (d, *J* = 6.5 Hz, 3H), 0.85–0.75 (m, 1H), 0.74–0.67 (m, 1H), 0.66 (s, 3H). ^13^C NMR (125 MHz, CDCl_3_) *δ* 212.47, 136.56, 116.00, 71.06, 56.30, 56.20, 54.09, 50.20, 49.86, 44.41, 42.55, 39.95, 38.77, 38.20, 36.42, 36.24, 35.75, 34.91, 31.78, 29.84, 29.35, 29.21, 28.28, 25.34, 24.16, 21.38, 20.80, 18.63, 12.77, 12.07.

*4-(((R)-6-((4S,5S,8S,9S,10R,13R,14S,17R)-4-allyl-10,13-dimethyl-3-oxohexadecahydro-1H-cyclopenta[a]phenanthren-17-yl)-2-methylheptan-2-yl)oxy)-4-oxobutanoic acid (****8****).* To a solution of compound **7** (100 mg, 0.23 mmol) in pyridine (10 mL) was added succinic anhydride (232 mg, 2.3 mmol) and DMAP (281 mg, 2.3 mmol) at room temperature. The reaction mixture was heated under reflux for 72 h. After cooling, the mixture was quenched with an aqueous solution of 2N HCl and extracted with AcOEt (10 mL × 3). The organic layer was washed with brine, dried over anhydrous Na_2_SO_4_, and concentrated. The residue was purified by silica gel chromatography (petroleum ether/AcOEt, 2/1, v/v) to give **8** (82 mg, 67%) as a white solid. ^1^H NMR (600 MHz, CDCl_3_) *δ* 5.82–5.71 (m, 1H), 5.05–4.92 (m, 2H), 2.62 (t, *J* = 6.8 Hz, 2H), 2.55 (t, *J* = 6.4 Hz, 2H), 2.50–2.37 (m, 2H), 2.33–2.28 (m, 2H), 2.26–2.21 (m, 1H), 2.02–1.96 (m, 2H), 1.83–1.77 (m, 1H), 1.74–1.69 (m, 3H), 1.65–1.60 (m, 1H), 1.59–1.48 (m, 2H), 1.42 (s, 6H), 1.38–1.33 (m, 7H), 1.28–1.20 (m, 2H), 1.20–1.10 (m, 3H), 1.09–1.07 (m, 1H), 1.05 (s, 3H), 1.03–0.93 (m, 2H), 0.90 (d, *J* = 6.5 Hz, 3H), 0.88–0.77 (m, 1H), 0.73–0.68 (m, 1H), 0.67 (s, 3H). ^13^C NMR (150 MHz, CDCl_3_) *δ* 212.65, 177.44, 171.35, 136.56, 116.03, 83.42, 56.31, 56.30, 54.08, 50.21, 49.87, 42.56, 41.19, 39.95, 38.79, 38.21, 36.25, 36.15, 35.70, 34.91, 31.78, 30.10, 29.84, 29.07, 28.29, 26.02, 25.99, 25.34, 24.17, 21.39, 20.43, 18.53, 12.78, 12.09.

*4-(((R)-6-((3R,4S,5S,8S,9S,10R,13R,14S,17R)-4-allyl-3-hydroxy-10,13-dimethylhexadecahydro-1H-cyclopenta[a]phenanthren-17-yl)-2-methylheptan-2-yl)oxy)-4-oxobutanoic acid (****9****).* By a similar procedure described for **LY295427**, compound **9** was obtained as a white solid (yield 87%). ^1^H NMR (500 MHz, CDCl_3_) *δ* 5.90–5.82 (m, 1H), 5.16–4.94 (m, 2H), 3.90 (d, *J* = 2.8 Hz, 1H), 2.62 (t, *J* = 6.3 Hz, 2H), 2.55 (t, *J* = 6.4 Hz, 2H), 2.31–2.18 (m, 1H), 2.01–1.91 (m, 2H), 1.83–1.77 (m, 1H), 1.73–1.64 (m, 4H), 1.60–1.53 (m, 3H), 1.51–1.45 (m, 3H), 1.42 (s, 6H), 1.38–1.30 (m, 6H), 1.28–1.19 (m, 3H), 1.17–1.10 (m, 2H), 1.09–0.96 (m, 5H), 0.90 (d, *J* = 6.4 Hz, 3H), 0.81 (s, 3H), 0.78–0.69 (m, 1H), 0.65 (s, 3H). ^13^C NMR (150 MHz, CDCl_3_) *δ* 177.38, 171.36, 138.32, 115.87, 83.43, 67.96, 56.57, 56.25, 54.43, 43.55, 42.49, 41.25, 40.23, 40.09, 36.51, 36.17, 35.71, 34.90, 34.36, 32.22, 32.12, 30.13, 29.09, 28.96, 28.29, 26.01, 25.99, 24.13, 23.84, 20.98, 20.40, 18.55, 12.36, 12.10.

*(R)-6-((3R,4S,5S,8S,9S,10R,13R,14S,17R)-4-allyl-3-hydroxy-10,13-dimethylhexadecahydro-1H-cyclopenta[a]phenanthren-17-yl)-2-methylheptan-2-yl (2-(3-(but-3-yn-1-yl)-3H-diazirin-3-yl)ethyl) succinate (****LY295427-probe****).* To a solution of compound **9** (95 mg, 0.17 mmol) in anhydrous dichloromethane (15mL) was added EDCI (100 mg, 0.52 mmol)、DMAP (64 mg, 0.52 mmol) and alkyne-diazirine-OH^2^ (72 mg, 0.52 mmol) at room temperature. The reaction mixture was stirred for 4 h at room temperature under a N_2_ atmosphere. Then, the reaction mixture was poured into water and extracted with dichloromethane (10 mL × 3). The organic layer was washed with brine, dried over anhydrous Na_2_SO_4_, and concentrated. The residue was purified by silica gel chromatography (petroleum ether/AcOEt, 10/1, v/v) to give **LY295427-probe** (71 mg, 61%) as a white solid. ^1^H NMR (600 MHz, CDCl_3_) *δ* 5.89–5.82 (m, 1H), 5.09 (d, *J* = 17.0 Hz, 1H), 5.01 (d, *J* = 10.1 Hz, 1H), 3.99 (t, *J* = 6.5 Hz, 2H), 3.90–3.88 (m, 1H), 2.61–2.53 (m, 4H), 2.28–2.24 (m, 1H), 2.05–1.92 (m, 5H), 1.83–1.76 (m, 1H), 1.74 (t, *J* = 6.4 Hz, 2H), 1.72–1.63 (m, 6H), 1.60–1.52 (m, 2H), 1.53–1.43 (m, 3H), 1.41 (s, 6H), 1.37–1.31 (m, 6H), 1.28–1.18 (m, 3H), 1.18–0.92 (m, 7H), 0.89 (d, *J* = 6.5 Hz, 3H), 0.87–0.82 (m, 1H), 0.80 (s, 3H), 0.77–0.69 (m, 1H), 0.64 (s, 3H). ^13^C NMR (150 MHz, CDCl_3_) *δ* 172.22, 171.36, 138.36, 115.82, 83.22, 82.55, 69.32, 67.84, 59.25, 56.57, 56.25, 54.44, 43.56, 42.49, 41.26, 40.25, 40.09, 36.52, 36.19, 35.71, 34.90, 34.38, 32.29, 32.24(2C), 32.13, 30.22, 29.27, 29.02, 28.29, 26.30, 26.03, 26.01, 24.13, 23.84, 20.97, 20.41, 18.56, 13.25, 12.36, 12.10. ESI−HRMS (m/z) [M+Na]^+^ calcd for C_41_H_64_N_2_NaO_5_, 687.4707, found 687.4704.

**Copies of ^1^H and ^13^C NMR spectra for the compounds**

**Compound 1:**

**^1^H NMR**


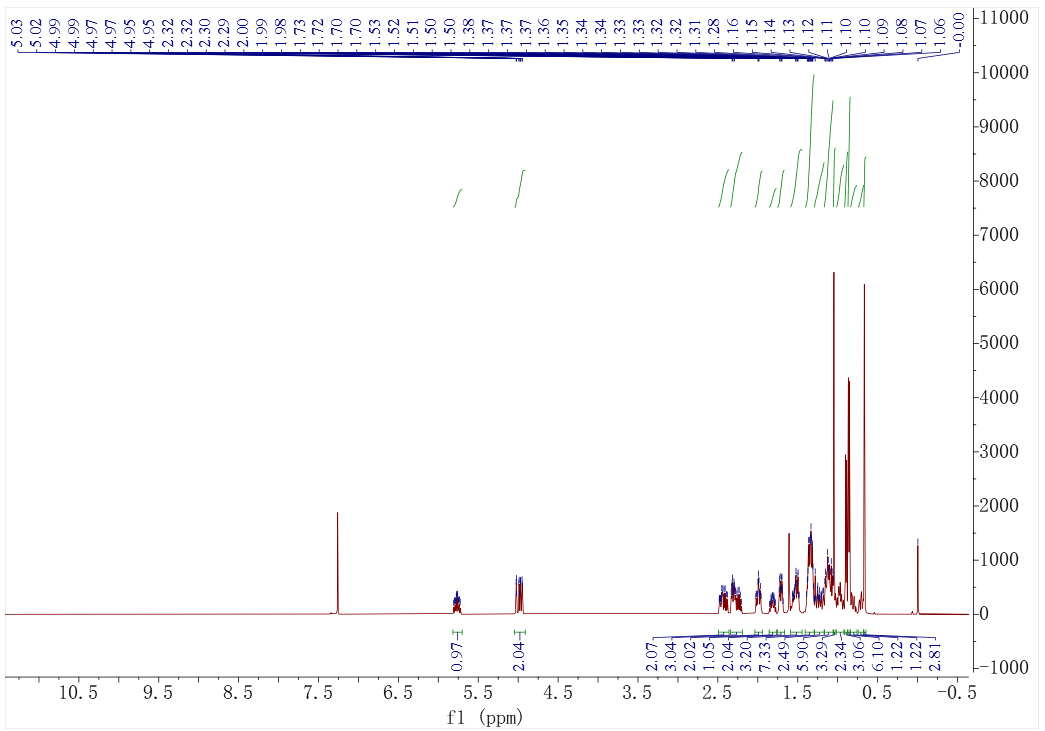


**^13^C NMR**


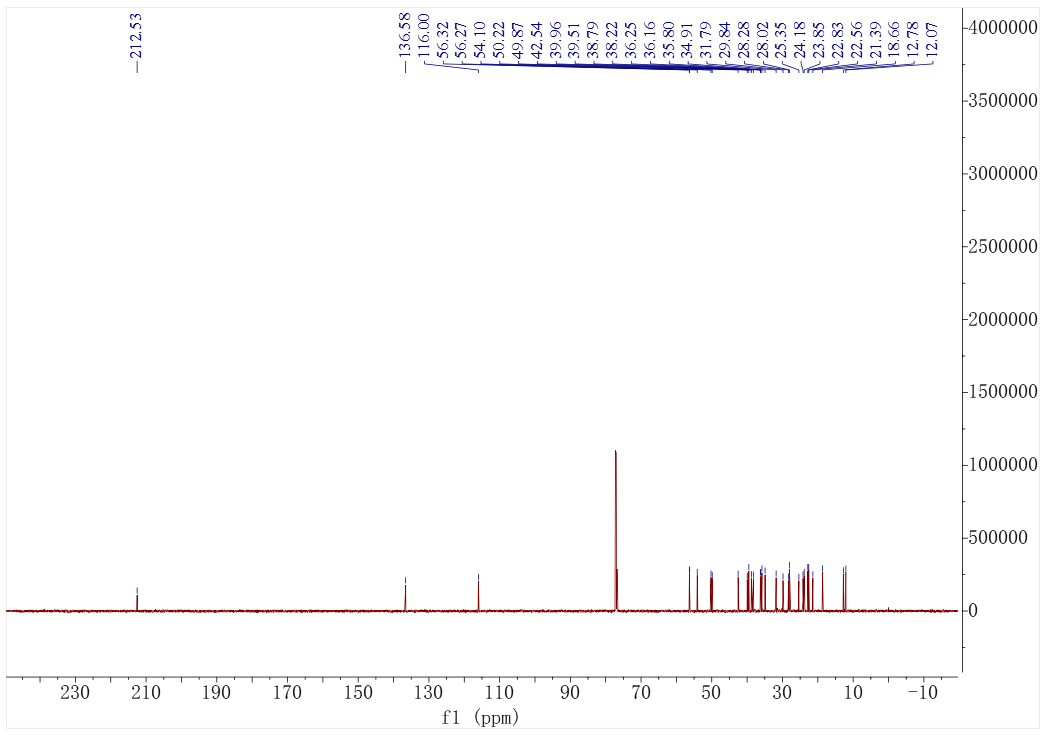


**LY295427:**

**^1^H NMR**


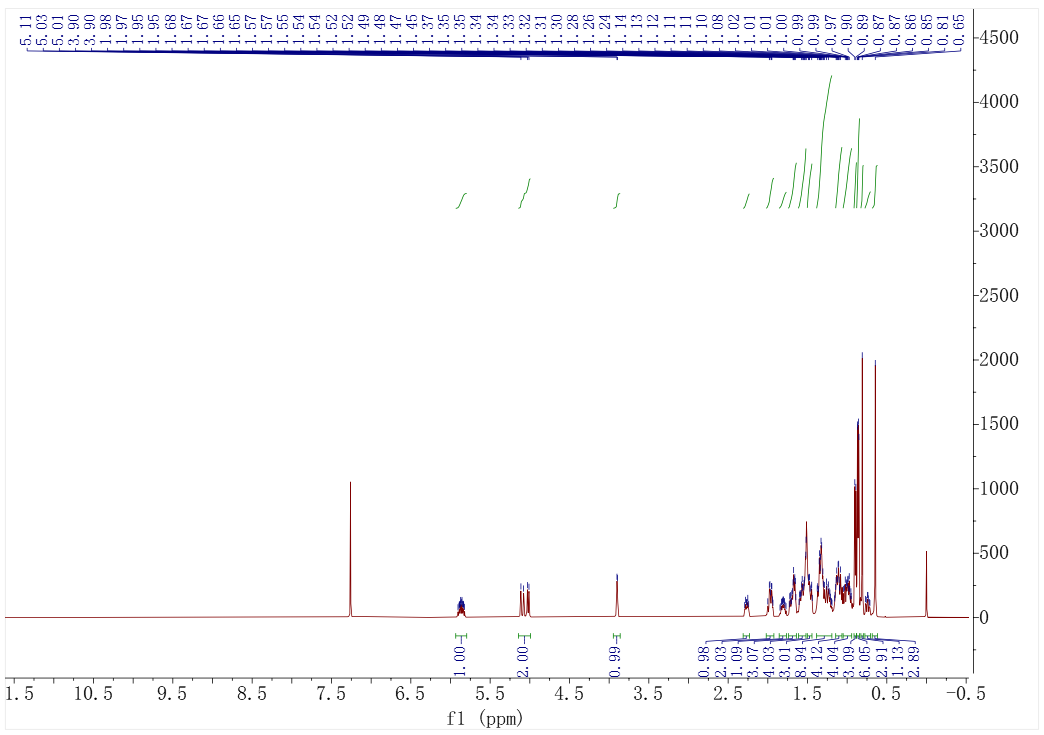


**^13^C NMR**


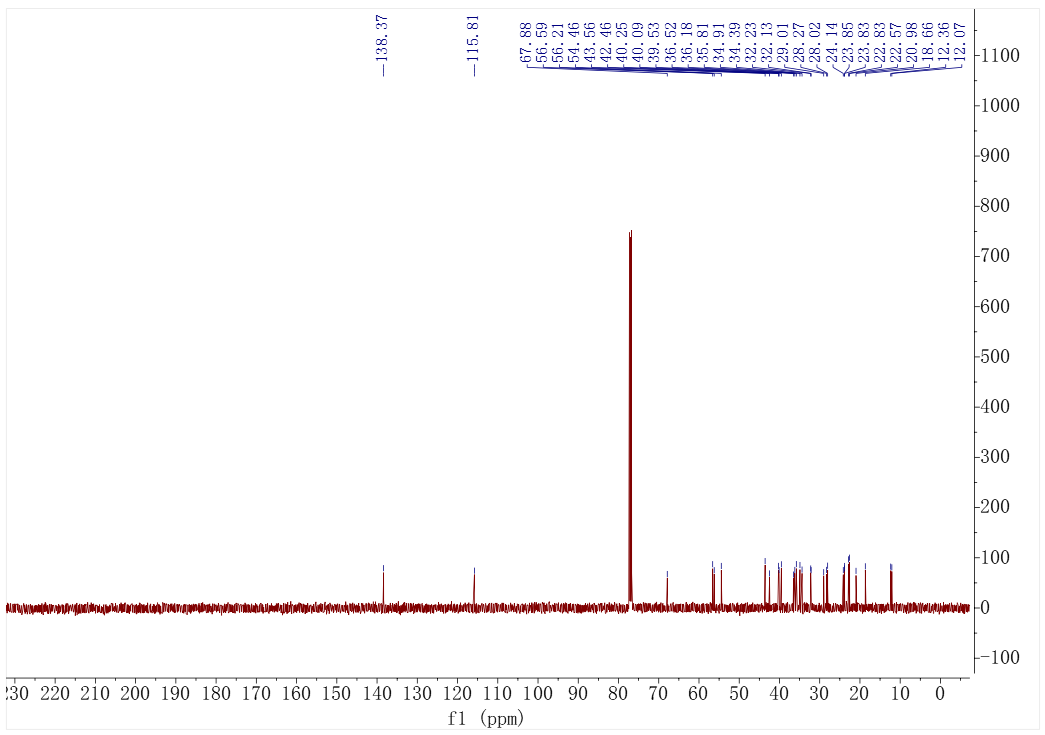


**Compound 2:**

**^1^H NMR**


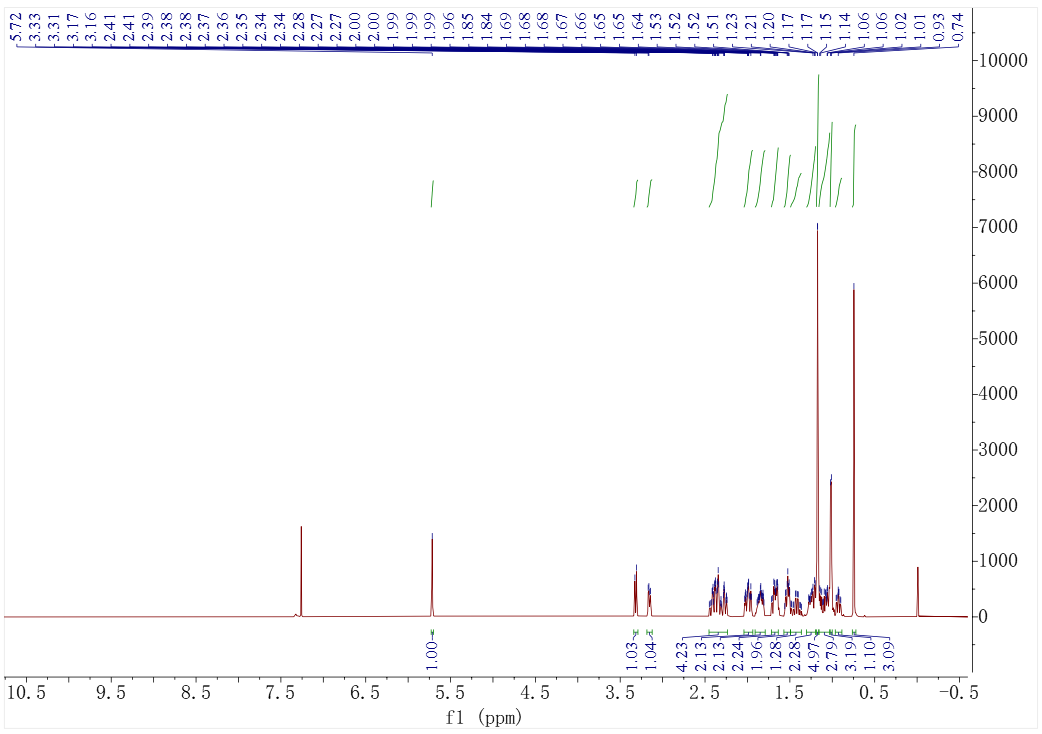


**^13^C NMR**


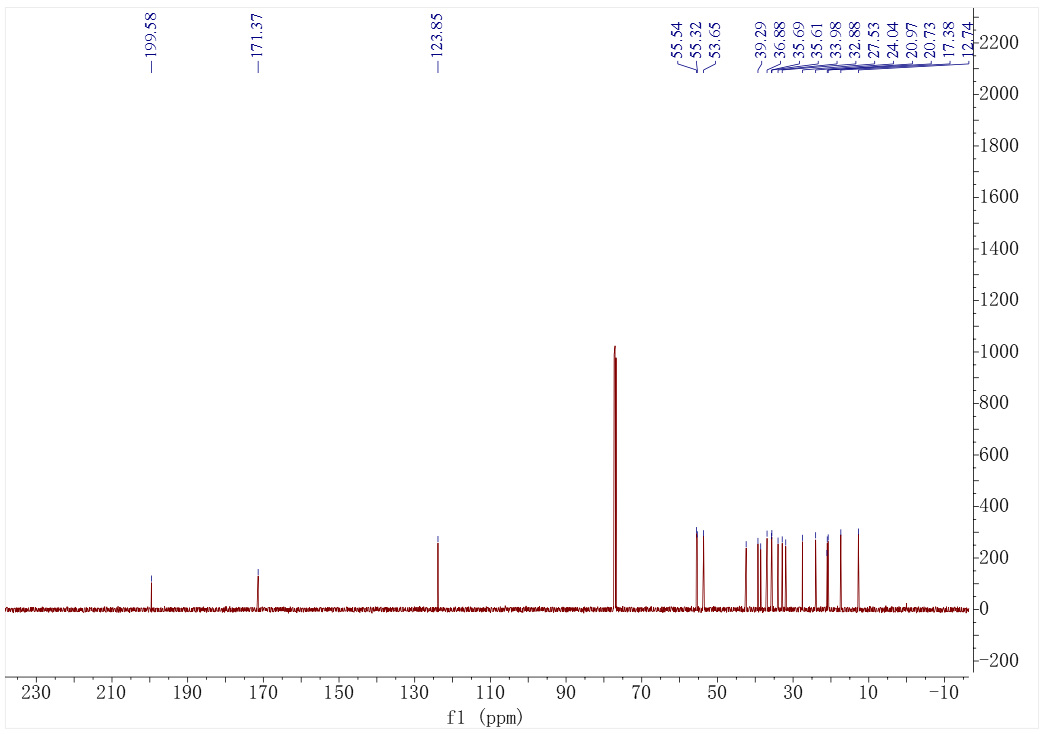


**Compound 3:**

**^1^H NMR**
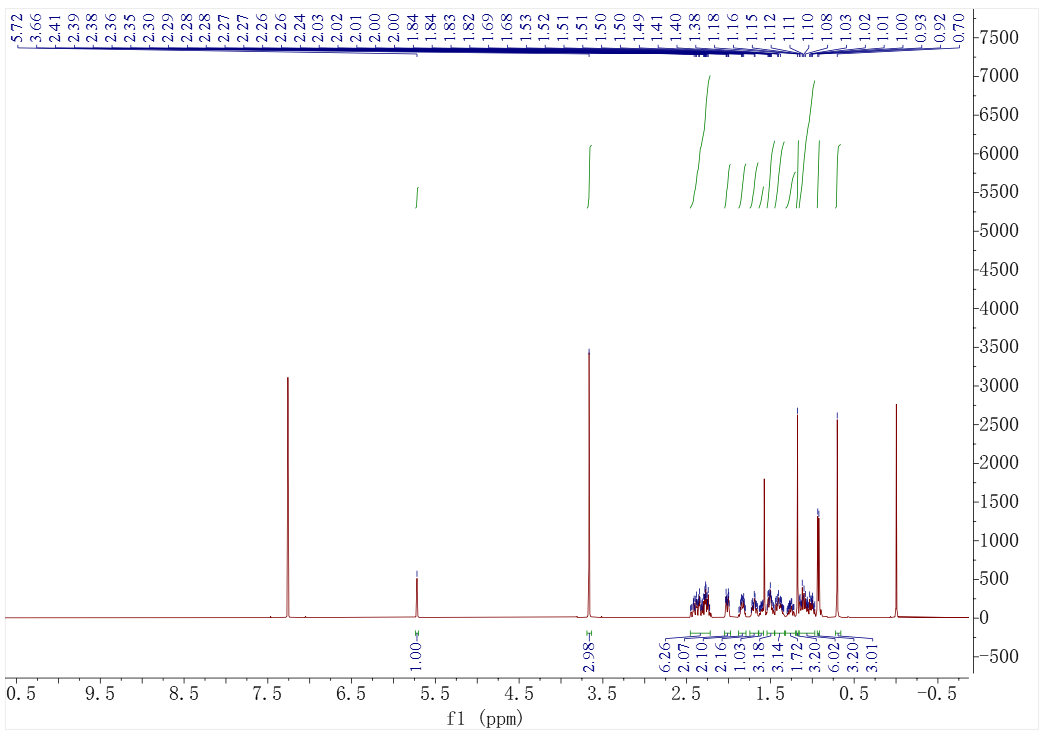


**Compound 6:**

**^1^H NMR**


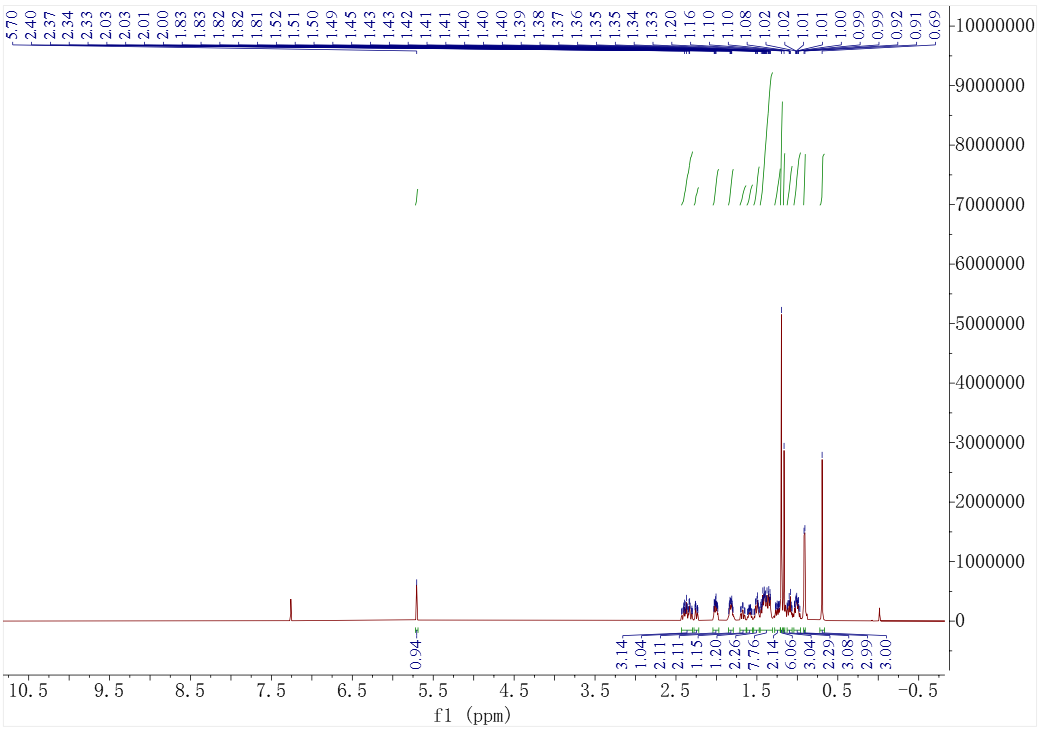


**^13^C NMR**


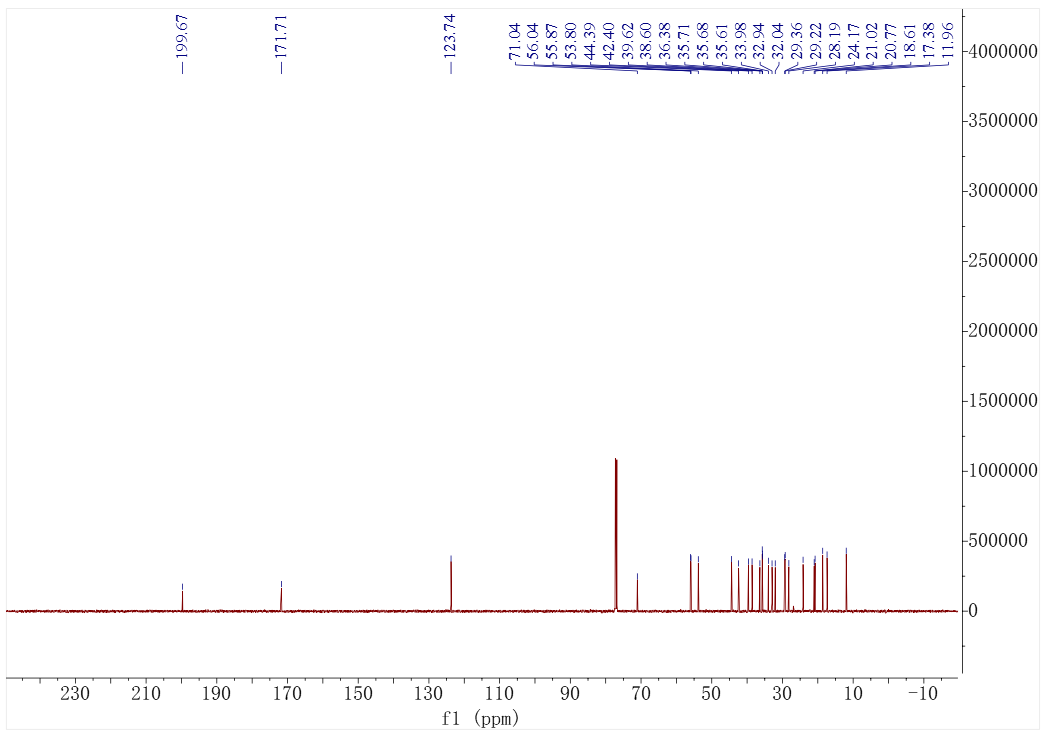


**Compound 7:**

**^1^H NMR**


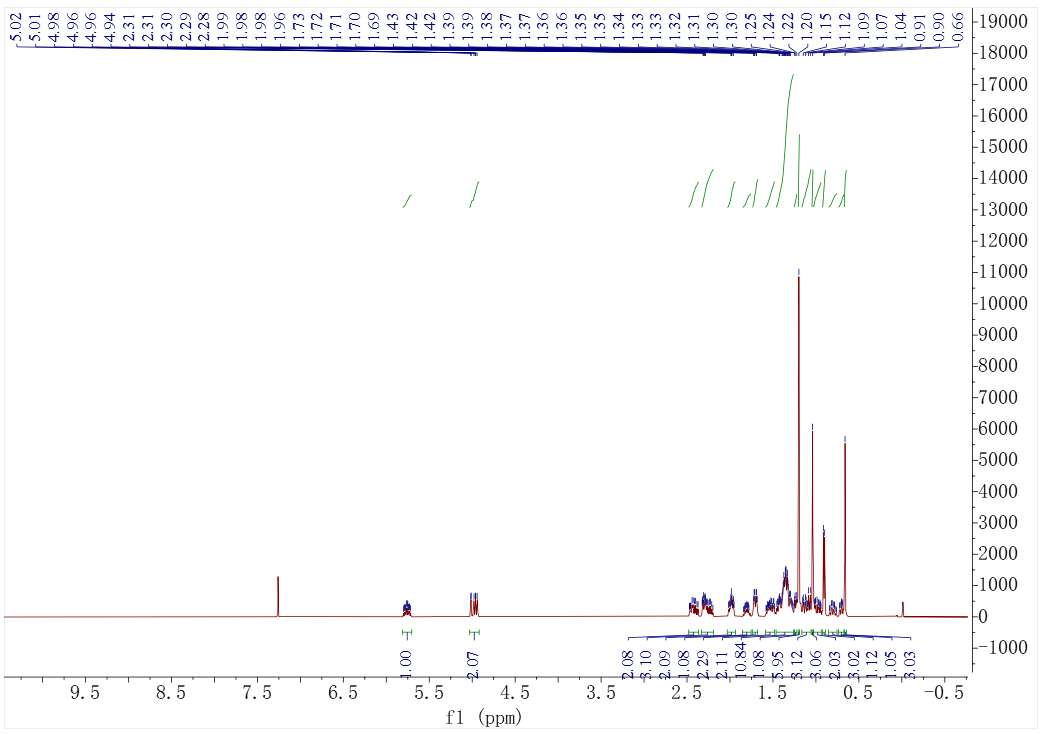


**^13^C NMR**


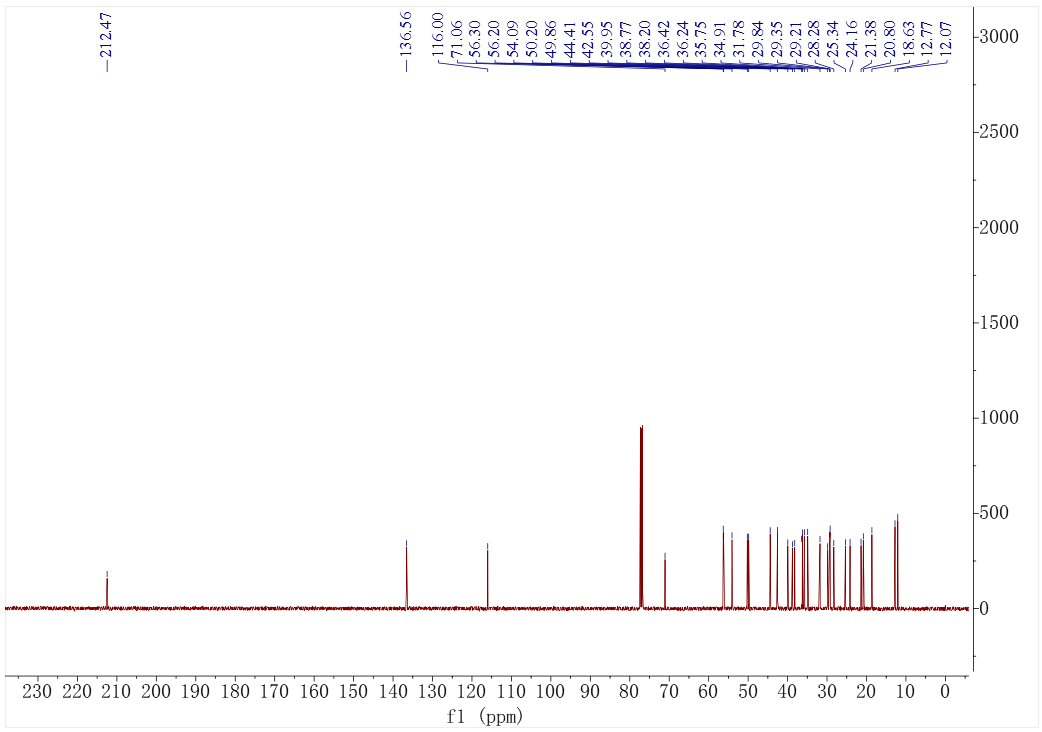


**Compound 8:**

**^1^H NMR**


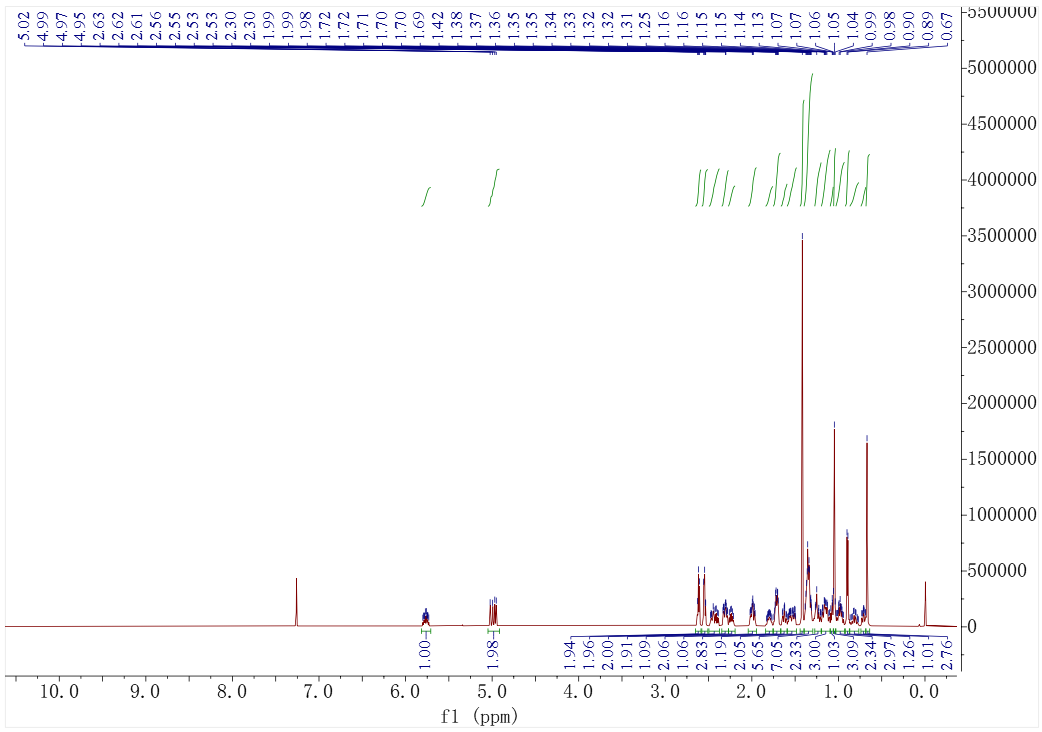


**^13^C NMR**


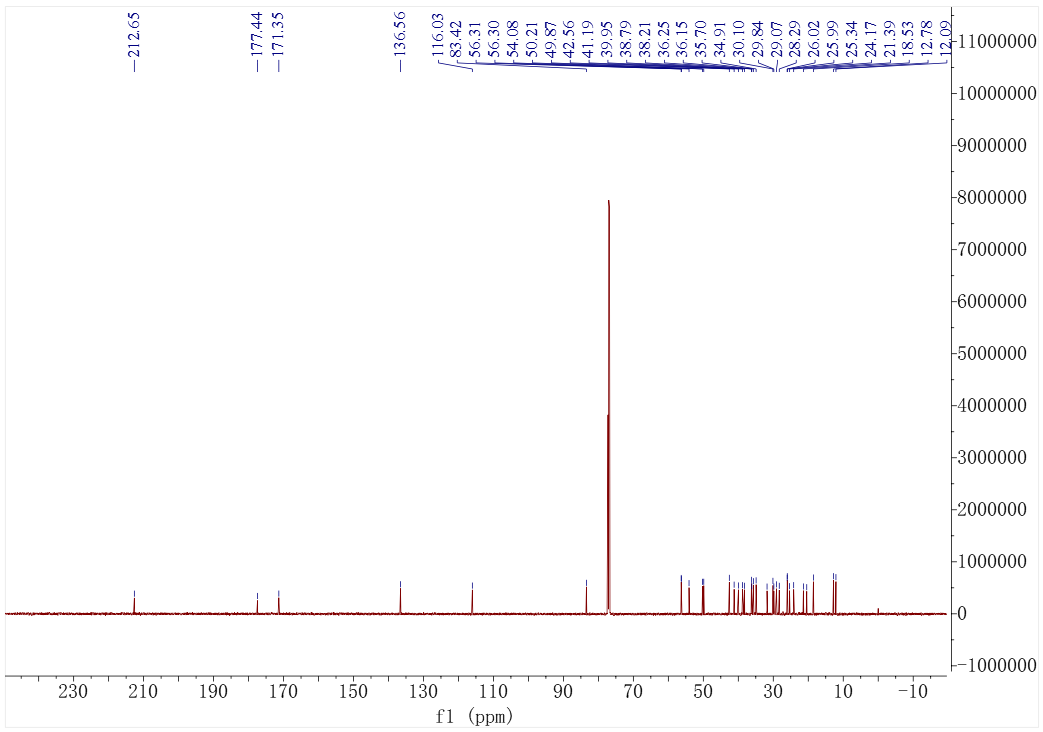


**Compound 9:**

**^1^H NMR**


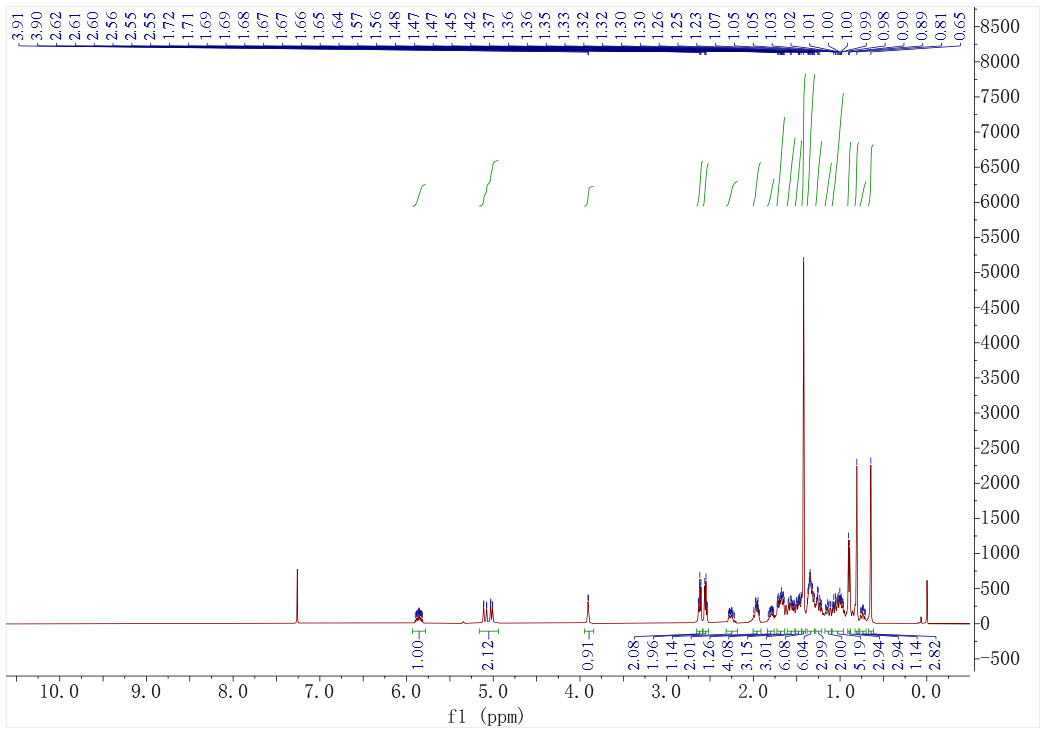


**^13^C NMR**


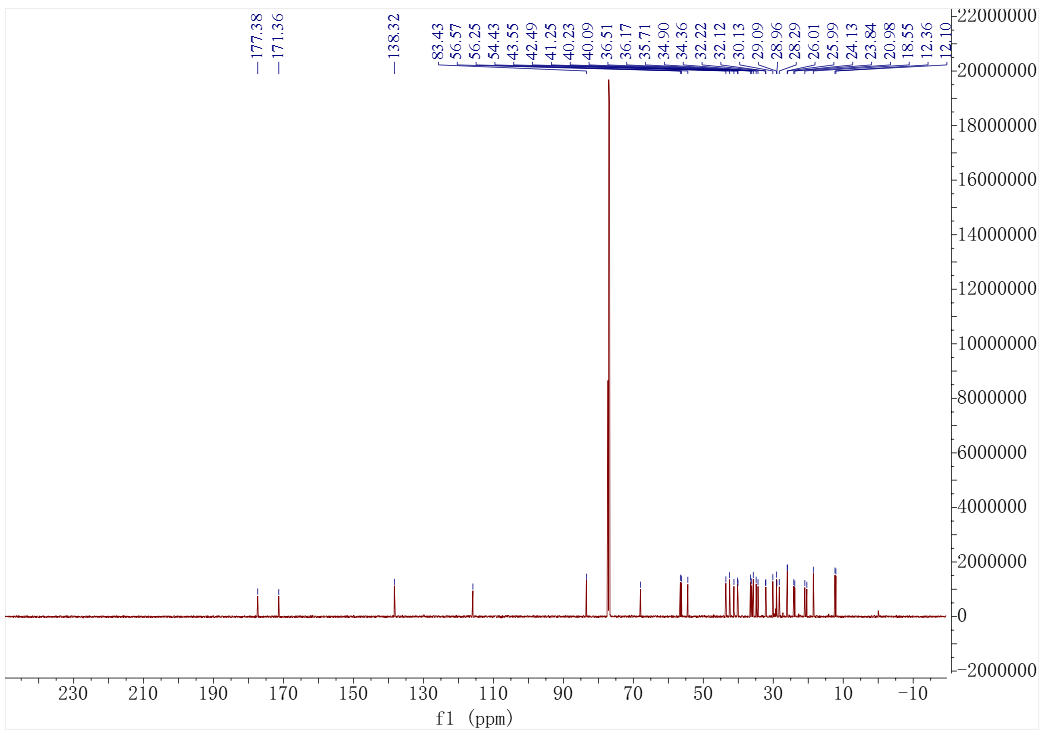


**LY295427-probe:**

**^1^H NMR**


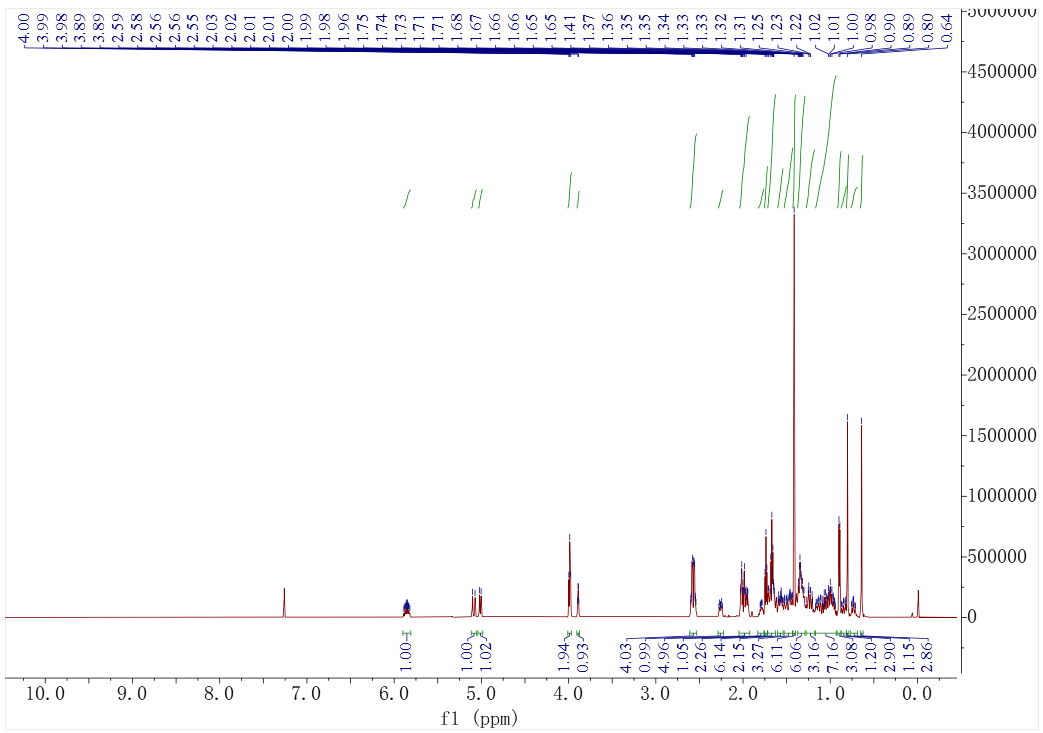


**^13^C NMR**


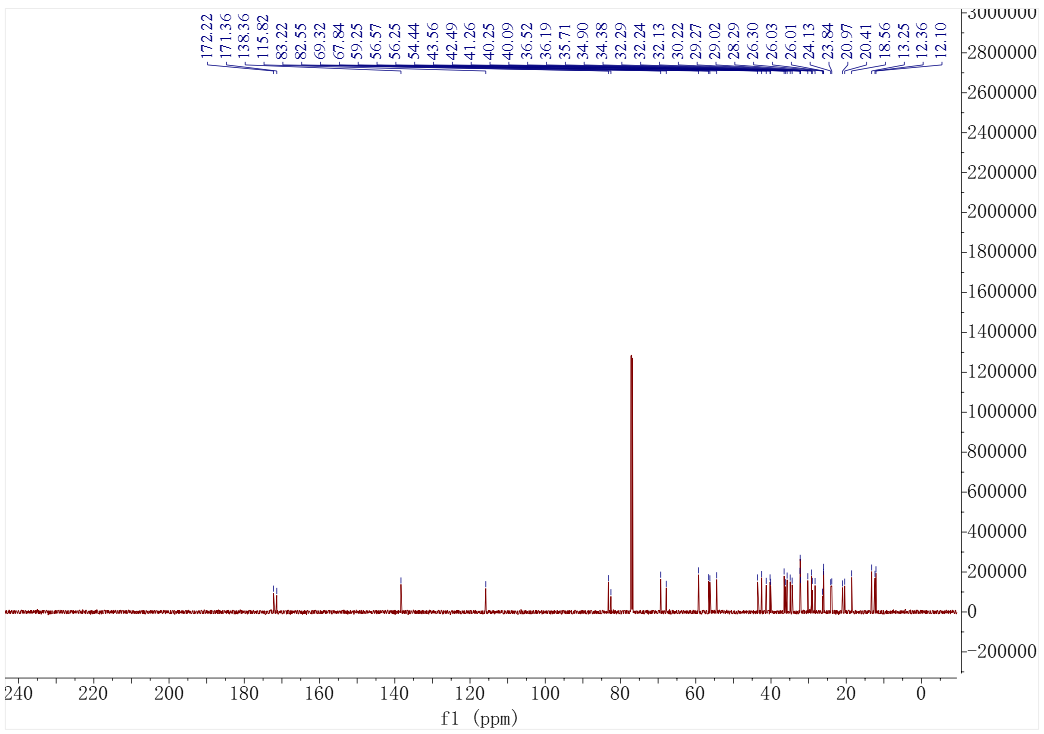


**HRMS**


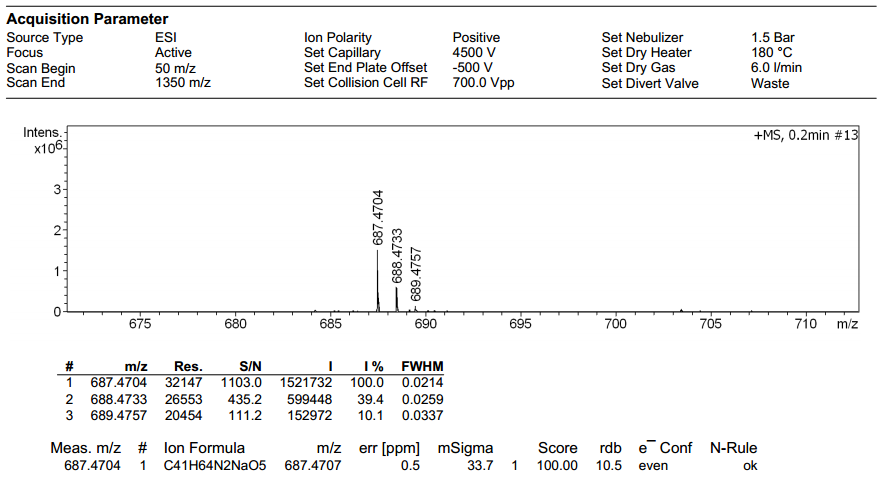

Supplement: Supplemental Figure [file mmc1.docx]
