# Supplementary material for: The compound LY295427 antagonizes 25-hydroxycholesterol through binding to INSIG
Source: J Lipid Res. 2026 Mar 6;67(4):101015. doi: 10.1016/j.jlr.2026.101015 (PMC13090600; doi:10.1016/j.jlr.2026.101015)
Supplement: Supplemental Table S1 [file mmc2.docx]

**Table 1. Primer sequences for real-time PCR.**

| **Primer sequences for real-time PCR.** | | |
| --- | --- | --- |
| **Species** | **Genes** | **Sequences of forward and reverse primers** |
| Hamster | Gapdh | GCAAGTTCAAAGGCACAGTCAA |
|  |  | CGCTCCTGGAAGATGGTGAT |
|  | Srebp2 | TAAGCAAGTACCTGGCGGTG |
|  |  | GCTTGTGATTGACCTGCTGC |
|  | Ldlr | AGACACATGCGACAGGAATGAG |
|  |  | GACCCACTTGCTGGCGATA |
|  | Hmgcr | AGATACTGGAGAGTGCCGAGAAA |
|  |  | TTTGTAGGCTGGGATGTGCTT |
|  | Lss | AGGGCACCAATGGATCACAG |
|  |  | GACCTGTGAAAGACGCAGGA |
|  | Fdft1 | AATCAGACCAGTCGCAGCTT |
|  |  | ACTCCAAGGAGATCGTGGGG |
|  | Insig1 | GGCTTGTGGTGGACATTCG |
|  |  | GGCGATGGTGATCCCAAGT |
|  | Hmgcs | CCTGGGTCACTTCCTTTGAATG |
|  |  | GATCTCAAGGGCAACGATTCC |
|  | Sqle | GCAGAGCCCAATGTAAGGGT |
|  |  | CTGCAACAACAGTCAACGGG |
|  | Dhcr24 | GGAAGGGTTGCTGTACTCCC |
|  |  | AGCTTGCTGGGTTCTACGTC |
|  | Nsdhl | ACCTATCCCAAGATGCAGCG |
|  |  | AGGCTACCCAGTAGGGGATG |
|  | Scap | CATCGGTATCTCCCTGGCAC |
|  |  | AGCAAGTCGGTCACCTGAAG |
|  |  | CTCTGTTCAGCTATTGGACGC |
